# The Versatility of Layered Two‐Dimensional Heterostructures for Energy Storage: Bridging Scientific Insights and Practical Applications

**DOI:** 10.1002/adma.202501490

**Published:** 2025-06-12

**Authors:** Neetu Bansal, Nitish Kumar, Prakash Kumar Pathak, Heejoon Ahn, Jing Tang, Yusuke Yamauchi, Rahul R. Salunkhe

**Affiliations:** ^1^ Materials Research Laboratory Department of Physics Indian Institute of Technology Jammu Jagti, NH‐44, PO Nagrota Jammu Jammu and Kashmir 181221 India; ^2^ Department of Industrial and Materials Science Chalmers University of Technology Göteborg SE‐412 96 Sweden; ^3^ Human‐Tech Convergence Program Department of Organic and Nano Engineering Hanyang University 222 Wangshimni‐ro, Seongdong‐gu Seoul 04763 Republic of Korea; ^4^ Shanghai Key Laboratory of Green Chemistry and Chemical Processes School of Chemistry and Molecular Engineering East China Normal University Shanghai 200062 China; ^5^ Department of Materials Process Engineering Graduate School of Engineering Nagoya University Nagoya 464‐8603 Japan; ^6^ Australian Institute for Bioengineering and Nanotechnology (AIBN) The University of Queensland Brisbane QLD 4072 Australia; ^7^ Department of Convergent Biotechnology & Advanced Materials Science Kyung Hee University 1732 Deogyeong‐daero, Giheung‐gu Yongin‐si Gyeonggi‐do 17104 South Korea

**Keywords:** 2D materials, anodes, energy storage, layered heterostructures, monovalent rechargeable batteries

## Abstract

Nanoscale manipulation of electronic and ionic charge interactions within electrode materials is the cornerstone for advancing electrochemical energy storage. Compared to bulk materials, 2D confined anodes provide lamellar channels to mobile ions for electrochemical interactions. However, individual 2D layers are often inefficient in delivering desired properties for stable and rapid kinetics in battery operations. To address this, 2D‐2D heterostructures (2D HRs) that integrate the properties of two or more layers via van der Waals or covalent bonds can give optimized interfacial features. These structures modulate electronic properties, such as band positions, activation energies, diffusion barriers, and binding energies for intercalating ions, thereby regulating the electrochemical characteristics of batteries to meet practical challenges. In this context, this review includes the latest experimental and theoretical investigations to explore the multifunctional roles of 2D HRs in monovalent ion (Li^+^, Na^+^, and K^+^) batteries (MIBs). First, it elucidates the fundamentals concerning the impacts of HRs in charge storage mechanisms and outlines pathways for synthesizing their novel designs. Then, it summarizes the different configurations of 2D HRs utilized in designing MIBs. Finally, it underscores the current challenges and future perspectives for implementing 2D HRs as advanced anode materials in batteries.

## Graphical abstract

This review highlights the potential of 2D‐2D heterostructures (HRs) in advancing monovalent ion‐based energy storage. It examines their role in tailoring charge interactions mechanisms, optimizing interfacial properties, and overcoming the limitations of individual layered materials. Covering synthesis methods, structural designs, and electrochemical advances, the review offers insights into design strategies and challenges, positioning 2D HRs as promising anode materials.

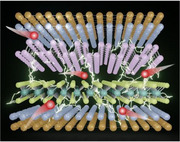

## 1 Introduction

**Figure 1:**
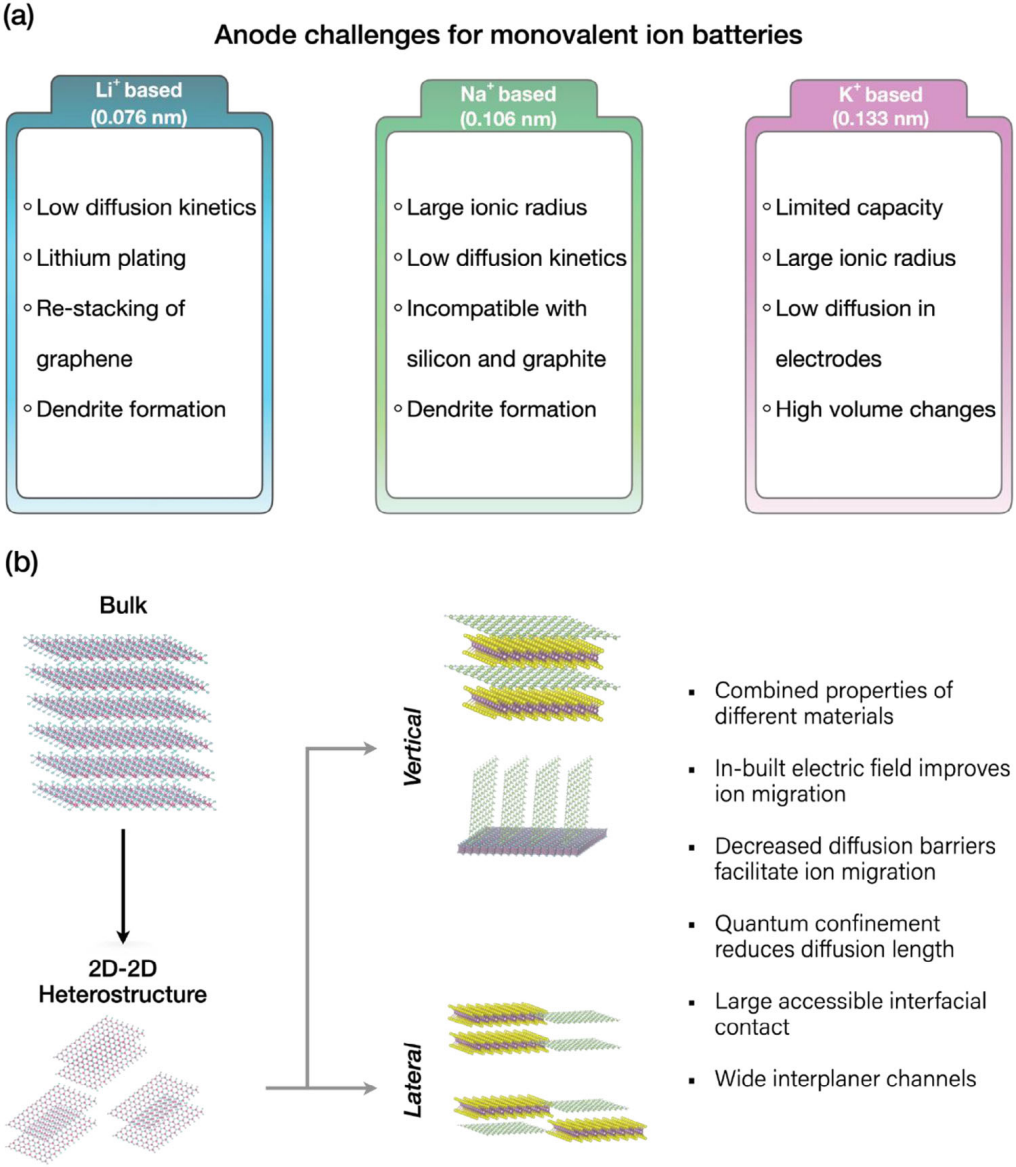
a) Schematic illustration highlighting the significant challenges for anodes in Li^+^, Na^+^, and K^+^ ion batteries. b) The diagram shows the transitions from bulk 2D structures to 2D HRs and their further classification into vertical and lateral HRs. The HR assemblies improve certain characteristics of anodes that help to address several challenges in batteries (mentioned by bullet points).

The rapid pace of global industrialization has dramatically accelerated the energy demand; hence, the fulfillment of energy storage devices (ESDs) has become an underlying issue. Electrochemical devices such as batteries, fuel cells, and supercapacitors are leading contenders in the market due to their distinctive attributes, like high energy and power density.^[^ ^1^ ^]^ Among these, batteries have become a massive part of the electrical industry and are anticipated to remain dominant in the foreseeable future.^[^ ^2^ ^]^ In particular, alkali metal or monovalent‐ion batteries (MIBs), including Li‐ion batteries (LIBs), Na‐ion batteries (SIBs), and K‐ion batteries (PIBs), owing to similar physicochemical properties, have garnered widespread attention at industrial and academic scales. The electrode and electrolyte materials primarily determine the energy storage performance of these battery systems. In this regard, the past two decades of 2003–2025 have witnessed the maturation of various anode/cathode materials and the rapid growth of new battery chemistries, some of which have evolved into viable choices for the battery industry.^[^ ^3^ ^]^ Nevertheless, each type of battery presents unique challenges, often arising from the interaction between metal ions (M^+^: Li^+^, Na^+^, and K^+^) and electrode materials (**Figure** **1**a). Typically, the larger size of Na^+^ (1.06 Å) and K^+^ (1.33 Å) ions brought major issues like sluggish diffusion and structural deformations that mitigate the stability of batteries. It is demonstrated that structural characteristics of anodes, such as atomic and molecular arrangement, interatomic distance, interfacial sites, and morphology, have an essential role in confronting these challenges.^[^ ^4^, ^5^ ^]^ Among these, morphological and interfacial features determine the wettability and interactions of active material with electrolytes. These characteristics precisely control the adsorption properties of M^+^ ions over the electrode, thereby regulating the capacity of batteries.^[^ ^6^ ^]^ In contrast, lattice structure parameters are strictly linked with the diffusion kinetics of M^+^ ions and their redox activity within the electrodes, which considerably impacts the stability and rate capability of batteries.^[^ ^7^ ^]^

The bulk materials often contain inaccessible interior sites for the ions and cannot be efficiently utilized, necessitating their structural modifications. One effective approach is to reduce the dimensionality of these materials, which significantly shortens ion diffusion pathways and facilitates deeper ion penetration. Since 2008, increasing attention has been directed toward confining anode materials to 2D nanostructures featuring atomically thin layered surfaces with a large surface‐to‐volume ratio and a mechanically flexible structure. Several 2D materials belonging to the family of graphite, transition metal oxides (TMOs), transition metal dichalcogenides (TMDs), phosphorene (P), transition metal carbides/nitrides (MXenes), and so on, with extraordinary properties, have attained significant acclaim in anode systems of MIBs.^[^ ^8^, ^9^, ^10^, ^11^, ^12^, ^13^ ^]^ The covalently bonded 2D layers are stitched together vertically by van der Waals (vdW) forces, which provide slit‐shaped channels and offer fast diffusion paths for mobile M^+^ ions in batteries. The dual‐sided surface of 2D layers multiplies the active sites and interaction with electrolytes. Such structural features reveal their popularity as anode materials for fast operations compared to their 3D counterparts.^[^ ^14^, ^15^, ^16^ ^]^ Each 2D material is impressively superior in terms of its inherited characteristics. However, no single 2D material provides all the essential properties crucial for the high performance of batteries, such as electronic conductivity, high interplanar spacing, and structural stability. For instance, TMDs with high redox activity can provide large specific capacities but suffer from poor structural stability during long‐term cycling due to irreversible redox reactions. In contrast, graphene (Gr), empowered with excellent electronic conductivity, reflects limited active sites that restrict its specific capacity. In addition, most 2D materials encounter poor cycling stability and rate capability, largely attributed to their tendency to aggregate over repetitive cycles and the development of polymeric gel‐like layers caused by electrolyte degradation during the electrochemical processes. Therefore, structural modulations that manipulate the chemical and electrical properties of these 2D architectures become necessary. To address this, several approaches, such as doping, heterostructure (HR) designing, and defect engineering, have been introduced that amplify the stability and performance of 2D materials.

Subsequently, HRs obtained by strategically hybridizing different materials with 2D layers can improve the performance of batteries. Depending on the dimensionality of foreign materials hybridized with 2D layers, the HRs can be categorized as 0D‐2D, 1D‐2D, 2D‐2D, and 3D‐2D types.^[^ ^17^ ^]^ Among these, 2D‐2D HRs (2D HRs) obtained by the synergy of different 2D offer uniform collaborative features over larger interfacial areas and can serve numerous locations for the ions compared to other configurations.^[^ ^18^ ^]^ Consequently, this unlocks an opportunity to simultaneously leverage the complementary properties of 2D materials belonging to different families.^[^ ^19^ ^]^ Moreover, the culminating physiochemical characteristics of HRs are highly determined by the intrinsic properties of each 2D layer (elemental composition, Fermi levels, lattice parameters, band gap, attached functional groups) and the synthesis process. After integrating the layers with different Fermi levels, the interaction between layers alters their electron density, electrical conductivity, diffusion barriers, mechanical strength, and adsorption energy for intercalating ions.^[^ ^20^, ^21^ ^]^ Therefore, the complementarity features of different layers can be optimized to provide the 2D HRs with a robust structure possessing less volume expansion, low charge transfer resistance, high redox activity, and a large accessible area to the electrolyte. For example, stacking of TMDs over Gr results in HR that showcases good conductivity (property of Gr) and redox active sites (property of TMDs).^[^ ^22^ ^]^ Overall, depending on the characteristics of each layer, the anode material can be optimized to achieve enhanced rate capability and specific capacity for batteries.

Furthermore, the 2D HRs can be constructed by knitting the 2D layers laterally by confining them in a single direction (lateral HR) or by stacking them vertically over each other (vertical HR) as a result of covalent or vdW forces (Figure 1b).^[^ ^23^, ^24^ ^]^ Several top‐down and bottom‐up synthesis methods have been unveiled for designing such 2D HRs.^[^ ^25^, ^26^ ^]^ In the last decade, this aspect has brought various potential composite materials into the energy storage field by utilizing diverse synthesis techniques. Despite their successful outcomes at the laboratory scale, none of these have proven to be scalable and feasible for industrial‐level battery fabrication. Hence, there is a need to find synthesis techniques to design HRs qualitatively on a large scale, which exhibit optimum performance for commercialization. As a result, this field requires significant attention from researchers and necessitates initial guidance for them to target 2D HRs for industrial battery applications.

To date, very few reviews have been published highlighting the valuable assets of 2D HR materials in ESDs.^[^ ^27^, ^28^ ^]^ However, a complete directional review combining the theoretical and experimental aspects of solely 2D HRs in batteries is still missing and needs significant attention. In this review, we present a systematic overview of the hybridization of different 2D layers for electrode materials in MIBs. First, we underscore the challenges for electrode materials in MIBs and the pivotal role of different 2D HRs in addressing these issues. In this regard, insight is provided into how interactions between M^+^ ions and 2D HR host materials can modify the physics and chemistry of the electrode/electrolyte interface, thereby contributing to the development of more stable battery devices. Furthermore, conventional and emerging synthesis approaches utilized for 2D HRs are briefly reviewed, and are compared in terms of cost, scalability, utility, and quality, which can add benign effects to achieve battery for the industry. We correlate the theoretically investigated fundamental properties of 2D HRs with the experimentally observed enhanced performances, statistically providing an overall evaluation of the electrochemical properties of the 2D HRs in batteries. Finally, we highlight the material selection criteria and strategies to boost the efficiency of 2D HRs as electrode materials. Overall, this review explores the multifunctional roles of 2D HRs in MIBs.

## 2 Advancements in 2D HRs

The progressive research on different chemistries of MIBs has facilitated the discovery of a wide range of anode materials. These research outcomes have led to the commercialization of certain electrode materials, such as graphite for LIBs and hard carbons for SIBs. The anode performance is influenced by numerous factors, including electrical conductivity, thermal stability, Gibb's free energy, structural characteristics, solvated ion properties, type of electrolyte, and type of interactions with ions. These properties collectively determine the practical suitability of any anode material. Depending on the interaction of ions, the electrochemical reaction mechanisms have broadly categorized the anode materials as (1) intercalation type, (2) conversion type, (3) alloying, and (4) organic compounds.^[^ ^29^ ^]^

The intercalation‐type materials include carbons (graphite, hard carbon, soft carbon) and some other compounds such as titanium or niobium‐based oxides (Li_4_Ti_5_O_12_, TiO_2_, Na_2_Ti_3_O_7_, Nb_2_O_5_, TiNb_2_O_7_) and MXenes. These materials generally have 2D layered structures or 3D frameworks, providing abundant lattice space for reversible movement of ions. The reversible reactions prevent the anodes from severe phase transformations and crystal structure damage, often resulting in prolonged cycling (≈5000 cycles). However, these materials exhibit limited active sites for ion storage, resulting in poor specific capacities (≈300 mAh g^−1^). These materials are cost‐effective and have received great acceptance in the battery industry.

In conversion‐type anodes, metal oxides (Fe_2_O_3_, Co_3_O_4_, NiO, MnO, MoO_3_, etc.), chalcogenides (MoS_2_, WSe_2_, CoS_2_, VS_2_, etc.), and phosphides (CoP, CuP_2_, NiP_2_) are majorly investigated materials. They follow certain reversible redox reactions to store the M^+^ ions, and the simultaneous multi‐electron transfer generates a high theoretical capacity for batteries. The reversible reaction is expressed as:

(1) $$\begin{array}{c} m_{a}y_{b}+\left(b*n\right)M^{+}+\left(b*n\right)e^{-}\;↔\;am+bM_{n}y\; \end{array}$$

where *m* represents the transition metals (*m*: Fe, Co, Ni, Cu, Mn, Mo, etc.) with *a* number of moles, *y* refers to nonmetallic anionic substances (O, S, P, Se, N) having *b* number of moles, while *n* indicates the valence state of *y*. The wide availability and tunability of *m* and *y* elemental compositions offer opportunities to obtain multiple electrochemical potentials. Nevertheless, the conversion reactions include continuous phase transformations and large‐volume changes that often lead to particle pulverization, voltage hysteresis, and electrical contact loss. This eventually causes their low coulombic efficiencies and fast capacity decay. Also, these materials inherit poor conductivity due to their semiconducting nature, which restricts their high‐rate performance.

Furthermore, alloy‐type anode materials include elements from the IVA and VA groups. These comprise metallic (Sn, Bi, Sb, etc.) and semimetallic (Si and Ge) elements. These elements (*m*) get alloyed with the multiple M^+^ ions (represented by *x*) during the charging–discharging process and, hence, are capable of performing with higher specific capacities than other anode materials (2–3 times that of conversion‐type anodes). The alloying process is represented as:

(2) $$\begin{array}{c} m+xM^{+}+xe^{-}\;\to M_{x}m \end{array}$$

However, the repeated alloying/dealloying reactions during the battery charge–discharge process often result in the formation of various irreversible and electrochemically inactive compounds. These byproducts induce substantial volume changes, structural pulverization, and even complete rupture of the electrode. The severity of these effects is further amplified in the presence of large‐sized Na⁺ and K⁺ ions. In addition, these nonuniformly formed compounds create heterogeneous active sites for the metallic ions, which is accompanied by massive dendrite growth and a rapid capacity decay within a few cycles (much higher than the conversion‐type materials).^[^ ^30^ ^]^ These materials also exhibit poor electrical conductivity, which further results in a drastic capacity fall at higher rates. Moreover, certain materials perform with dual reactions, i.e., by combining more than one phenomenon. For example, some layered chalcogenides, namely, Bi_2_S_3_, Sb_2_S_3_, and SnS_2_, first undergo a conversion reaction and then follow an alloying reaction that brings together the merits and drawbacks of each method.^[^ ^22^ ^]^ Similarly, there are some metal chalcogenides, such as MoS_2_ and FeS_2_, that initially follow intercalation phenomena and then show redox conversion reactions.

The inorganic materials in the above‐mentioned compounds contain strong covalent/ionic bonds that mainly cause structural deformations during the continuous charge–discharge process. In contrast, organic materials, including small molecules and large polymeric chains, are composed of weak interactions that provide sufficient space for reversible ionic movements. This can help to achieve high‐rate batteries. In addition, the environmental friendliness, sustainability, and diversity are the main highlights of these materials. Several organic compounds attain C═O, N═N, and C═N bond sites, which enhances redox activity.^[^ ^29^ ^]^ Despite their high capacity, flexible structure, and easy synthesis control, the low conductivity and tendency to dissolve in electrolytes showcase their unsuitability for commercial batteries.

The limitations associated with each type of anode demand effective processing approaches for materials. Also, their 3D bulk structures restrain the accessibility of ions, resulting in their partial utilization in electrodes. Confining them to one direction produces 2D‐type materials, which mitigates the issue of limited ionic accessibility. Since the discovery of Gr in 2004, dimensionally confined 2D materials have gained huge attention. After 2010, such layered materials have been widely recognized as promising electrode materials in LIBs.^[^ ^31^, ^32^ ^]^ Their covalently bonded atomically thin layered architecture significantly enhances performance due to the distinct advantages, including reduced diffusion length, mechanical flexibility, high surface‐to‐volume ratios, and slit‐shaped channels for efficient ion transport.^[^ ^16^ ^]^ It is well known that ion diffusion time (*τ*) in the electrode is proportionally related to diffusion length (*L*) by the relation τ∝ *L* ^2^/*D (D is diffusion coefficient, mainly dependent on the material and temperature)*.^[^ ^33^ ^]^ This implies that the 2D structured materials with a shorter diffusion length allow faster and deeper penetration of ions compared to 3D bulk materials. This consequently enhances the charging and discharging rates of batteries. Furthermore, the advantages of 2D structures for ion diffusion in batteries can be described by Fick's second law, stated as:^[^ ^34^ ^]^

(3) $$\begin{array}{c} \;\frac{\partial C}{\partial t}=D\;\frac{\partial^{2}C}{\partial x^{2}}\; \end{array}$$

where $\frac{\partial C}{\partial t}$ represents the change of M^+^ ion concentration (*C*) with time *t*, *D* denotes the diffusion coefficient, and $\frac{\partial^{2}C}{\partial x^{2}}$ is the derivative of the concentration gradient with respect to position *x*. At a given time, the ion diffusion in solid electrodes depends on the concentration gradient of the ions.^[^ ^35^ ^]^ Attributing to the short diffusion pathways of 2D materials, the electrodes can respond to different concentrations of ions more quickly ($\frac{\partial C}{\partial t})$, thereby boosting the rate performance of batteries.^[^ ^36^ ^]^ In addition, the increased, $\frac{\partial^{2}C}{\partial x^{2}}$ due to the thin structure shows that the ion distribution over the surface of electrodes is more uniform, leading to less polarization during the charging and discharging of batteries.^[^ ^37^, ^38^ ^]^

For the practical adaptability of any 2D electrode material with high electrochemical activity, it must possess chemical, mechanical, and thermal stability under extreme conditions. Despite the immense merits of 2D materials, they encounter limitations when utilized individually as electrode materials in batteries. For instance, Gr and MXenes offer excellent conductivity and mechanical stability but are prone to restacking and cracking issues.^[^ ^15^ ^]^ Weak interlayer forces and a thin structure make them highly fragile, which increases the complications in the manufacturing process. Conversely, TMOs and TMDs, while offering robust redox activity and potential for high specific capacities, are hampered by structural instabilities and poor conductivity. In addition, a number of materials, such as MXenes, silicene, and black phosphorene (BlackP), are chemically unstable in air and oxidize with time at certain temperatures, making their storage highly challenging for large‐scale applications. Also, when their oxidized counterparts are used as electrode materials, they often exhibit poor performance in batteries. To survive the real‐world stresses of humidity and temperature instability, key engineering strategies need to be introduced. **Table** **1** unveils the comparative overview of the strengths and challenges associated with different classes of 2D materials, which helps to identify the issues associated with different materials that can degrade battery performance.

**Table 1:** A comparative analysis of the strengths and limitations of various 3D materials and their 2D counterparts for guiding the selection of compounds to achieve 2D HRs with tailored properties.

3D materials	2D counterpart	Merits	Limitations
Graphite	Gr	Flexible, high electrical and thermal conductivity, large surface area	Moderate capacity, the charge stored only on the surface, restacking, low wettability
TMDs	TMDs	High theoretical capacity, low operating potential, open layered structure, active edge sites	Fast capacity drop due to conversion reactions, and low conductivity
MAX ceramics	MXene	High electronic and ionic conductivity, hydrophilic, high mechanical strength	Restacking occurs, thermodynamically metastable, low capacity, less active sites
TMOs	TMOs	High electrochemical activity, low cost	Low electronic conductivity, high volume expansion due to conversion reaction, low ionic diffusion
TMOs	LMHs	High electrochemical activity	Hydroxides not compatible with the nonaqueous electrolytes (not suitable for nonaqueous batteries), poor conductivity
Germanium	Germanene	High theoretical capacity	Difficult to synthesize freestanding material
Silicon	Silicene	High theoretical capacity	Difficult to synthesize freestanding material
Phosphorous	P	High theoretical capacity, electric conductivity, surface area, adjustable bandgap	Large volumetric changes, extremely reactive to oxygen

Note: Gr **=** graphene, TMDs = transition metal dichalcogenides, TMOs = transition metal oxides, LMHs = layered metal hydroxide, P = phosphorene.

**Figure 2:**
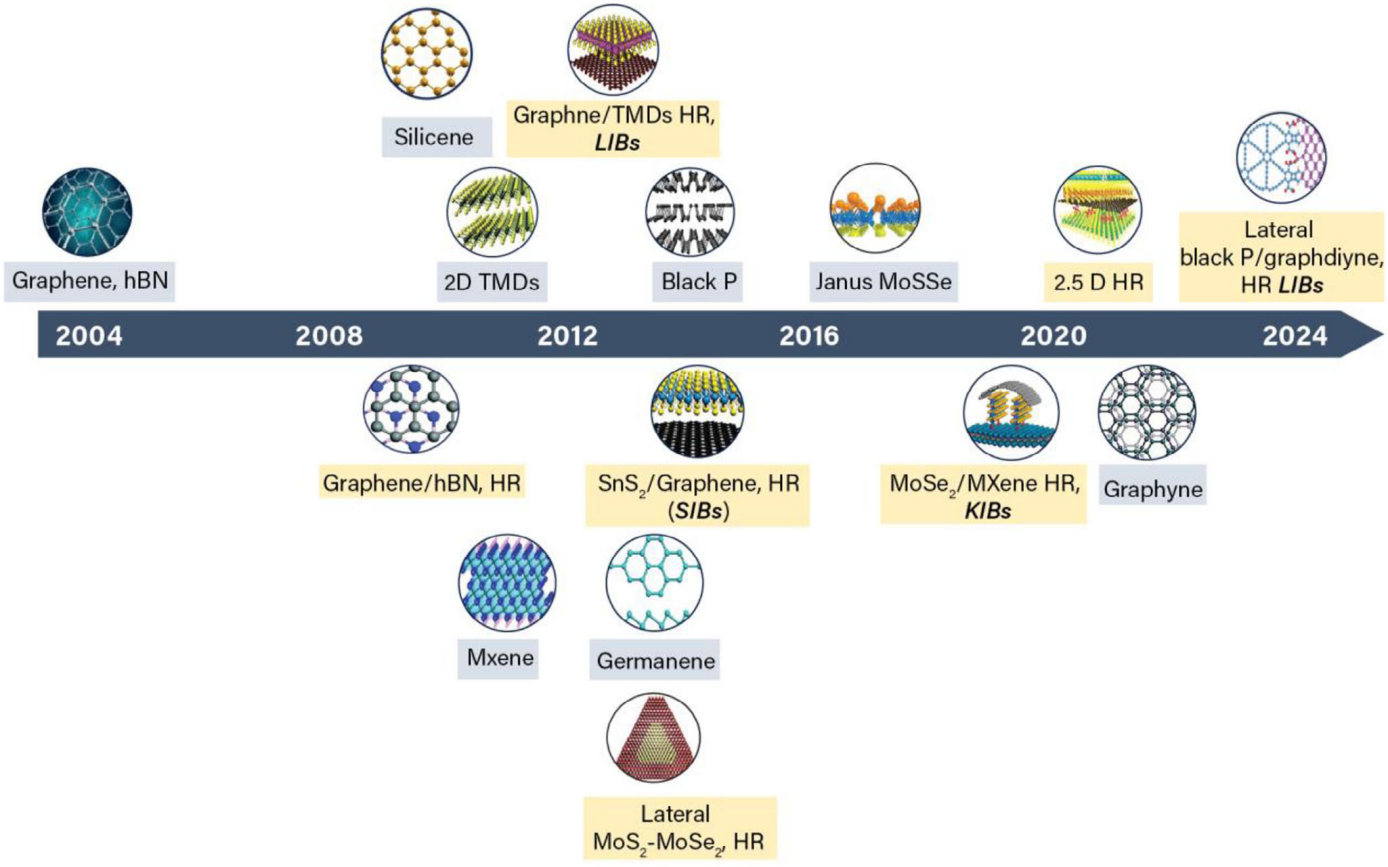
The timeline of various 2D lattices (grey boxes) and 2D HRs (yellow boxes) with different configurations. The discovery of 2D lattices led to a surge in the development of 2D HRs. Integrating various 2D lattices into a HR enhances the overall properties of a single superlattice, making it a promising anode material for batteries.

To circumvent these challenges, 2D HRs have been developed strategically by hybridizing the complementary properties of diverse 2D layers, thereby improving battery performance. The intrinsic properties of different 2D materials (e.g., the conductivity of Gr and the redox activity of TMOs) are integrated to form 2D HR with optimal electronic conductivity, interplanar spacing, and structural stability for battery electrodes. Nonetheless, the interfacial coupling between different layers plays a crucial role in determining the stability of the electrode material and the performance of batteries. Notably, in simple composites, the interfacial coupling is significantly weak, often resulting in sluggish kinetics of M^+^ ions and aggregation of active materials, ultimately leading to a capacity fade and rapid rate decay.^[^ ^39^ ^]^ However, in HRs, a strong force of interaction bridging the different layers provides an indestructible structure to the electrode. For example, electrostatically synthesized HR of Li*_x_* V_2_O_5_·*n*H_2_O and reduced graphene oxide (rGO) with strong interactions exhibit better rate performance (≈120 mAh g^−1^ @ 0.2 A g^−1^) than the physically mixed composite material, where it reaches 0 mAh g^−1^.^[^ ^40^ ^]^ In this context, the formation of covalently bonded HRs is more favorable for stabilizing the performance of batteries.^[^ ^39^ ^]^ Depending on the interactions between different 2D stacks, these are classified as vertical (vdW/covalent interactions) and lateral HRs (covalent interactions) (Figure 1b). For assembling lateral HRs, the layers should exhibit similar lattice structures. Therefore, TMDs such as MoS_2_, WS_2_, and SnS_2_ with minimal lattice mismatch are highly investigated for such HRs.^[^ ^41^ ^]^ Although strict lattice matching is not required in the vertical assembly of 2D HRs, vdW interactions can provide 2D HRs with incoherent lattice matching. Such structures sometimes lead to Moirés superlattices.^[^ ^42^ ^]^ Consequently, it is imperative to identify the crystal structures of different layers before assembling them in HR. **Figure** **2** illustrates the advancements in developing different lateral and vertical 2D HRs alongside the discovery of 2D materials. It also presents some of the benchmarks of 2D HRs in monovalent batteries.

## 3 Fundamentals of Stable 2D HRs for Battery Applications

**Figure 3:**
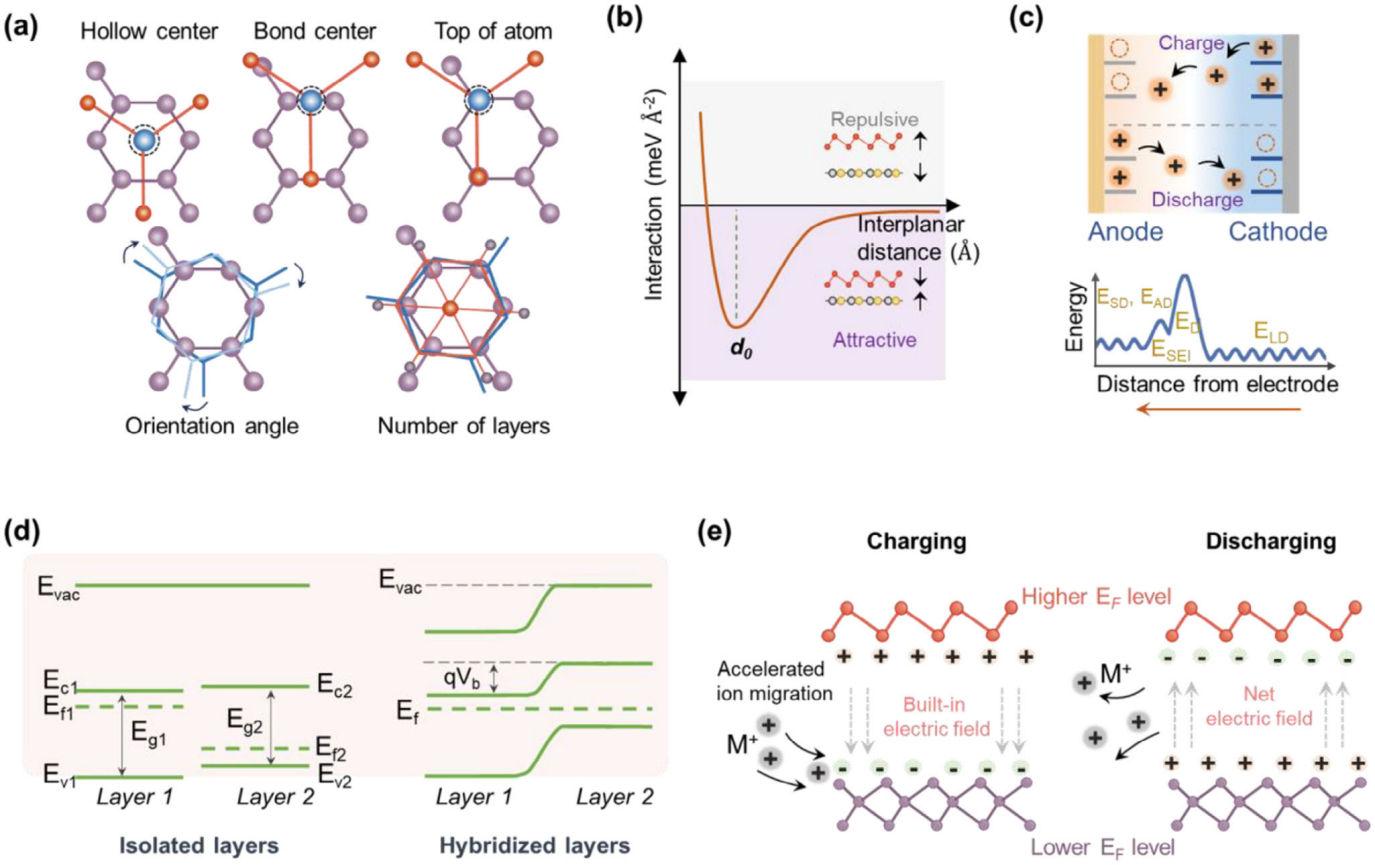
a) The illustration of different arrangements of overlapping layers that can affect their performance as electrodes for batteries. The configurational characteristics depend on the position of atoms concerning other layers (hollow center, bond center, and top of atom), the angle of orientation between layers, and the number of 2D layers participating in HR. b) The plot depicts the variation in interlayer interactions as a function of interplanar distance. c) Schematic illustrating the rocking chair mechanism of monovalent M^+^ ion batteries and corresponding energy barriers encountered by M^+^ ion during intercalation. (*E_LD_*: liquid diffusion, *E_D_*: desolvation energy, *E_AD_*: adsorption energy, *E_SEI_*: SEI layer crossing, and *E_SD_*: solid diffusion in electrode). d) Energy band diagram for isolated layers with different Fermi levels (*E*_f1_ and *E*_f2_) and their hybridized layered HR with combined Fermi level (*E*_f_′) and corresponding built‐in potential difference (*V*_b_). e) Schematic of built‐in electric field model in hybridized 2D HR during charging and discharging processes.

For the rational design of efficient 2D HRs, it is crucial to comprehend their interfacial properties and their interactions with mobile M^+^ ions. Obtaining the HRs with a stable lattice structure for the anodes is essential, as it decides several parameters such as active sites, steric hindrance, and reversible mass transfer for consistent battery performance. In this regard, we initially highlight the parameters utilized to identify the robust HR configurations, mainly dependent on the lattice structure and orientation of each layer. The thermodynamically stable configuration of HRs can be obtained by calculating their formation energy (*E_F_*) which is defined as:^[^ ^43^ ^]^

(4) $$\begin{array}{c} E_{F}=E_{HR}\;-\;\sum_{i=1}^{n}E_{i}\; \end{array}$$

where *E_HR_* is the total energy of HR, and *E_i_* represents the total energies of *i*th monolayer (*n* is the number of different monolayers participating in 2D HR). The HR with higher negative values of *E_F_* will be more stable.^[^ ^43^ ^]^ However, it is noteworthy that the stacking configurations, atomic positions, number of layers, and interplanar spacing of different layers highly influence *E_F_*. Considering this, some of the possible arrangements of layers in 2D HRs influencing the *E_F_* are represented in **Figure** **3**a. Typically, for the different configurations or arrangements of InSe over Gr (hexagon hollow center, bond center, and top of carbon atoms), illustrated in the top three images of Figure 3a, the HR attained dissimilar values of *E_F_* (−0.06869, −0.06171, and −0.06128 eV, respectively).^[^ ^44^ ^]^ Among these, the hexagon center with a high negative *E_F_* value comes out to be the most stable configuration. Furthermore, to find the most stable configuration of Ti_2_CS_2_ and blue phosphorene (BlueP) HR, Yuan et al. rotated the BlueP layers around Ti_2_CS_2_ at different angles, 0°, 60°, 120°, 180°, 240°, and 300° to get six distinct configurations.^[^ ^43^ ^]^ Among these, the HR with the 120° angle attains the highest negative energy (*E_F_* = −1.27 eV), signifying the most stable configuration. Using a similar approach, several other stable HRs have been identified by calculating their *E_F_* values.^[^ ^45^, ^46^, ^47^ ^]^

Moreover, for long‐term reversible ionic diffusion and large accessible active sites in HR, optimum interactions between the layers must exist. If the interactions are too weak, the layers may delaminate during cycling; if too strong, the reduced interlayer spacing can hinder ionic movement. The area‐averaged binding energy of HR (*E_B_*) serves as a useful parameter to estimate the strength of adhesion between different layers after they are brought together. The equation for *E_B_* is given as:^[^ ^23^ ^]^

(5) $$\begin{array}{c} \;E_{B}=\frac{\left(E_{HR}-\;E_{layer1}-E_{layer2}-E_{layer3}-\text{…}\;\right)}{A} \end{array}$$

where *A* is the interfacial area of layers, *E*_*layer*1_, *E*_*layer*2_, *E*_*layer*3_, and so on represent the total energy of mutually independent single layers fixed in the corresponding HR lattice.^[^ ^23^ ^]^ In addition, the *E_B_* is also calculated with respect to per atom.^[^ ^48^ ^]^ The *E_B_* of HRs at the equilibrium distance (*d_o_*) between layers gives an idea about the type of interaction (covalent/ionic/vdW) between them. Figure 3b illustrates the plot for *E_B_* versus interplanar distance (*d*) between layers. At larger *d* values, the force of interaction is very weak, whereas at lower *d* values, it is repulsive, which makes the HR unstable. The low *E_B_* of HR at the *d_o_*, such as −14.6 meV Å^−2^ for InSe/Gr;^[^ ^44^ ^]^ −41 meV per C atom for Ti_2_CF_2_/Gr HR, −16.67 meV Å^−2^ for InSe/MoS_2_,^[^ ^49^ ^]^ and −20 meV Å^−2^ for borophene/Gr^[^ ^50^ ^]^ (Bph/Gr) demonstrates the optimal interactions corresponding to the equilibrium state. Therefore, such flexible and stable 2D HRs are expected to be highly suitable for the long‐term intercalation‐deintercalation of ions in batteries.^[^ ^51^ ^]^

After achieving a stable configuration of 2D HR, the interaction of M^+^ ions with electrode materials plays a significant role. MIBs generally operate via a rocking chair mechanism while charging–discharging, as depicted in Figure 3c. The moving M^+^ ions encounter various energy barriers during ionic diffusion toward the electrodes. These include the energy associated with diffusion in the liquid electrolyte (*E_LD_*) corresponding to interaction with solvent molecules, desolvation energy (*E_D_*), crossing the solid electrolyte interphase (SEI) layer (*E_SEI_*), adsorption at the electrode surface, and diffusion into the solid crystal electrode material as shown by energy versus distance plot in Figure 3c.^[^ ^52^ ^]^ Among these, the adsorption energy (*E_AD_*) and the solid diffusion energy (*E_SD_*) are the parameters that primarily depend on the structure of the active material and their atomic interaction with M^+^ ions.^[^ ^42^ ^]^ Therefore, lowering these barriers is critically essential for the higher charge transfer capability of anodes. Numerous theoretical investigations have demonstrated how coupling 2D layers with different configurations results in the fundamental physical, electronic, and chemical modulations (e.g., charge redistribution, separation, transport, work function, and lattice distortion) in HRs. This leads to several intrinsic phenomena that are beneficial for enhancing the performance of batteries. This section outlines these phenomena, methods of observing them, and their role in improving the rate capabilities and stability of batteries.

### 3.1 Built‐in Electric Field

It is well known that the electrochemical activity across electrodes is enhanced in proportion to their charge transfer capabilities. Depending upon the position of the Fermi level and bandgaps, the 2D layers possess semiconducting (WS_2_, MoS_2_), semimetallic (Gr, MXenes), or insulating (hBN) properties.^[^ ^53^ ^]^ In context to this, the 2D HRs with distinct chemical compositions and variable physical properties can be integrated, in which both layers either belong to the same family (TiS_2_/MoS_2_, WSe_2_/SnSe_2_, Gr/MXene)^[^ ^54^, ^55^ ^]^ or different families (Gr/WSe_2_, MoS_2_/hBN, etc.).^[^ ^56^, ^57^, ^58^ ^]^ When a heterojunction is formed between the layers with different Fermi energy levels or work functions, the rearrangement of bands occurs to minimize the system's energy, causing band bending. In the process, the electrons migrate from the layer with a higher Fermi level (n‐type) toward the layer with a lower Fermi level (p‐type), resulting in a potential difference (built‐in potential, *V_b_*) between the energy levels of both sides.^[^ ^59^ ^]^ The complete process of band rearrangement is illustrated by a schematic of staggered‐type HR in Figure 3d. Such a potential difference generates a “built‐in electric field (BIEF)” from the layer with a higher Fermi level to the layer of a lower one, as displayed in Figure 3e.^[^ ^60^ ^]^ Consequently, the BIEF develops opposite charges on the layers (positive charge over the layer with a high Fermi level and negative charge on the layer with a low Fermi level). The positively charged M^+^ ions get directed toward the layer with an excessive negative charge by the strong coulombic force between them.^[^ ^61^, ^62^ ^]^ As a typical example, in MoS_2_/graphdiyne oxide (MoS_2_/GDYO) HR, density functional theory (DFT) simulations demonstrated that the work function difference between MoS_2_ (5.84 eV) and GDYO (5.53 eV) leads to 0.23 e charge transfer from GDYO to MoS_2_, facilitating Li^+^ ionsn PD toward the interface.^[^ ^63^ ^]^ This drives the faster diffusion of ions to the inner layers of the electrode, thereby increasing the utilization of active mass. Besides such theoretical investigations, BIEF can be examined by employing scanning Kelvin probe microscopy (SKPM), which determines the difference in surface potential.^[^ ^64^, ^65^ ^]^ In a study for SIBs, TiNbO_5_ and rGO were electrostatically deposited over a Si wafer, and using SKPM, the potential difference between them was estimated to be 50 mV, providing evidence for some induced electric field.^[^ ^65^ ^]^

Furthermore, to understand the interfacial electron transfer pathway near the Fermi level of HR, the density of state (DOS) plots are obtained by DFT calculations. Relative to the single structured 2D material, the 2D HR attains more electron density near the Fermi level, indicating the improved conductivity of the material.^[^ ^66^ ^]^ Projected DOS (PDOS) of 2D HRs can imply the interaction between its layers. Due to the presence of Ti atoms in metallic MXene layers on both sides of VSe_2_, the electron density near the Fermi level of VSe_2_ becomes higher, depicting the excellent electron mobility of 1T‐VSe_2_‐MXene HR.^[^ ^67^ ^]^ The peak overlap in PDOS plots indicate the strong interaction between the atoms of the layers.^[^ ^68^ ^]^ Moreover, Bader charge analysis quantifies the charge transfer, which can be known by the charge density difference (Δ*ρ*) [Equation (6)] at the HR interface.^[^ ^69^, ^70^ ^]^

(6) $$\begin{array}{c} \Delta \rho =\rho_{HR}-\rho_{layer\;1}-\;\rho_{layer\;2}-\;\text{…} \end{array}$$

where *ρ_HR_*, *ρ_layer 1_*, and *ρ_layer 2_* are the electron densities of the HR, layer 1, and layer 2, respectively. Typically, considering the example when BlackP is loaded by the MXene layer, it was observed that the charge density around P atoms changed in the range of −0.5 to −0.9.^[^ ^70^ ^]^ It indicates that the BlackP can now easily intake the electrons from Na, facilitating the fast sodiation process.

The rapid ion transfer also leads to improved rate capability of the active material. For instance, when MoSe_2_ was hybridized with FeSe for SIBs, its specific capacity at a high rate (10 A g^−1^) improved from ≈260 to 427.1 mAh g^−1^, attributed to the BIEF developed by heterogeneous structures.^[^ ^71^ ^]^ Similar investigations have been accomplished for LIBs and PIBs.^[^ ^22^, ^62^, ^72^ ^]^ Interestingly, the rapid movement of ions prevents the clustering of alkali ions and improves sluggish diffusion kinetics, which elevates the capacity and cyclability of batteries. However, an intriguing phenomenon accounts for the reversal of BIEF during discharging. In HR, the layer with high reaction reversibility triggers the de‐sodiation process, which causes it to be Na^+^ deficient compared to the other layer. The Na^+^ difference between layers yields an induced electric field generally opposite to the initial direction, as observed for MoS_2_/Fe_9_S_10_ HR by computing Gibb's free energy changes (∆*G*).^[^ ^73^ ^]^ The Fe_9_S_10_ with the ∆*G* value of −1.49 eV shows better sodiation/desodiation reversibility than MoS_2_ (−2.37 eV). Therefore, Fe_9_S_10_ delivers Na^+^ ions faster than MoS_2_, thereby producing the Na^+^ ion deficit surface of Fe_9_S_10_ and the Na^+^‐rich surface of MoS_2_, which leads to the reversed field (Figure 3e).^[^ ^64^, ^73^ ^]^ Therefore, the optimal selection of each layered structure is crucial to generate significant BIEF for improved reversible diffusion kinetics.

### 3.2 Adsorption Energy

During the intercalation process, after crossing the SEI layer, the M^+^ ion first adsorbs over the surface of the electrode material. *E_AD_* is the energy of interaction of M^+^ ions with the atoms of the anode material without bonding with them, which helps to identify the foremost structures of anodes. The higher negative *E_AD_* (NAE) values of each ion are representative of the better affinity of the ion with the electrode material and increased BE values. In this case, the electrode surface uniformly adsorbs a larger number of ions and eventually enhances specific capacities. The following equation can compute the *E_AD_* of ions in HR:^[^ ^74^, ^75^ ^]^

(7) $$\begin{array}{c} E_{AD}=\frac{E_{HR+nM^{+}}-\;E_{HR}-nE_{M^{+}}}{n}\; \end{array}$$

where *E_HR_* is the total energy of pristine HR (no guest ions attached), $E_{HR+nM^{+}}$ is the energy when *n* number of M*^+^* ions are adsorbed, and $\;E_{M^{+}}$ is the total energy of an isolated M^+^ ion.

**Figure 4:**
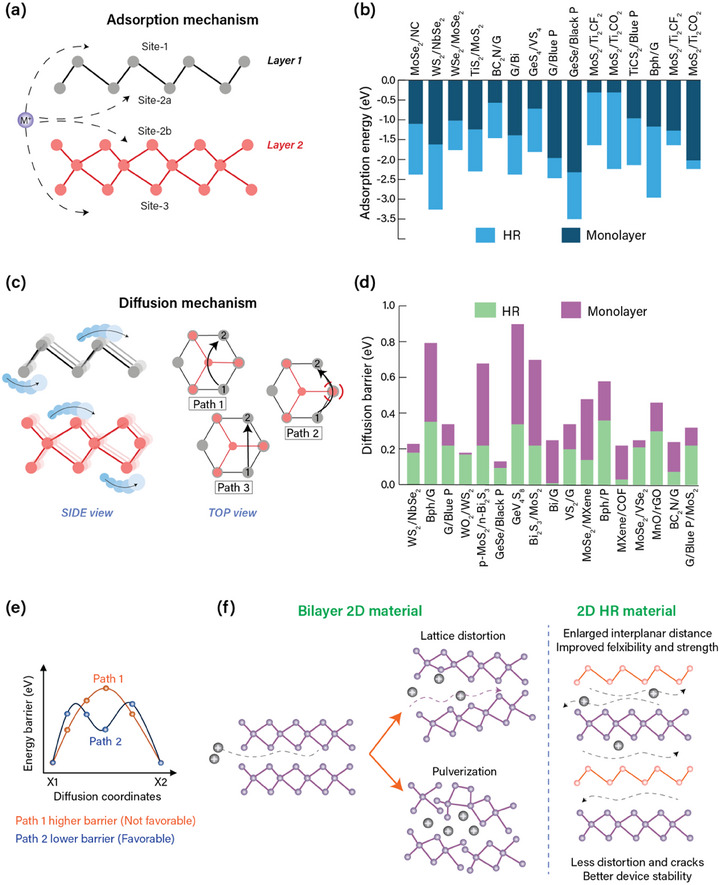
The schematic illustration of a) the adsorption mechanism and b) the stacked bar plot comparing the adsorption energies^[^ ^43^, ^50^, ^55^, ^78^, ^84^, ^85^, ^86^, ^87^, ^88^, ^89^, ^90^, ^91^, ^92^ ^]^ of different 2D layers and corresponding 2D HRs. c) The schematic represents the diffusion mechanism of M^+^ ions along different paths, and d) the stacked bar plot represents the decreased diffusion barriers for 2D HRs than their 2D counterparts.^[^ ^45^, ^50^, ^75^, ^85^, ^87^, ^88^, ^89^, ^90^, ^94^, ^98^, ^99^, ^100^, ^101^, ^102^, ^103^ ^]^ e) The *E_SD_* barrier for M^+^ ions at different diffusion coordinates across two paths (Path 1 and Path 2). Path 2, with a lower energy barrier, is favorable for the diffusion of ions. f) Schematic illustration of lattice changes (distortion and pulverization) observed in bilayered 2D materials during diffusion of ions. The 2D HRs with enlarged interplanar spacing and high mechanical strength help mitigate these lattice changes.

In the practical adsorption mechanism, the M^+^ ion can target different sites in the HR electrode. Typically, for the HRs consisting of two distinct layers, the ions can migrate toward the top surface (site 1) of layer 1, the bottom of layer 2 (site 3), and the middle side of both layers (site 2a/site 2b), as shown in **Figure** **4**a.^[^ ^43^ ^]^ Furthermore, depending on the properties of distinct atoms belonging to different layers, an HR can have numerous adsorption sites. (For example, 32 sites for Li^+^ ions in Bph/Gr HR, 22 sites for Li^+^ ions in Bph/MoS_2_ HR).^[^ ^50^, ^76^ ^]^ However, among these, only a few sites (exhibiting low *E_AD_*) are energetically favorable for adsorption.^[^ ^77^ ^]^ Analogously, Li et al., using DFT calculations, verified this phenomenon for two types of HRs: MoS_2_/Ti_2_CF_2_ and MoS_2_/Ti_2_CO_2_.^[^ ^78^ ^]^ In both the HRs, ions acquire maximum NAE when they are under the effect of both layers, compared to when they are on top or bottom of the 2D HR, where they face the impact of monolayer counterparts. This shows that the ion is likely to adsorb at the site where it sees the effect of both layers, and hence, HRs bring stability to ion adsorption.^[^ ^77^, ^78^, ^79^ ^]^

Furthermore, the characteristics of (1) M^+^ ions and (2) atoms decide the affinity and type of bond (ionic, covalent, or vdW) between them. The stronger affinity suggests that the M^+^ ions are no longer loosely available, preventing dendrite formation and self‐discharge in electrolytes.^[^ ^76^ ^]^ Thus, it is a vital aspect to be highlighted since it directly affects the number of ions stored and, hence, the capacity of batteries. Yuan et al. compared the adsorption behavior of different M^+^ ions in Ti_2_CS_2_/BlueP HR.^[^ ^43^ ^]^ The HR was loaded separately on nine different locations by each M^+^ ion (Li^+^/Na^+^/K^+^). The results showed that the Li^+^, Na^+^, and K^+^ ions acquired different stable sites; the Li^+^ ion was adsorbed into the HR interface, while Na^+^ and K^+^ ions onto the Ti_2_CS_2_‐side surface. This is attributed to the large radius of Na^+^ and K^+^, which significantly increased the interlayer distance *d*, making the *E_AD_* more negative (Na^+^: −2.126, and K^+^: −2.634 eV) on the Ti_2_CS_2_‐side surface than that at the interface (Na^+^: −1.270 and K^+^: −1.765 eV).^[^ ^43^ ^]^ Increased interlayer distance may decrease the influence of hybridization of layers and is expected to make dangling layers that are unstable for the adsorption of ions. In addition, the number of ions loaded also affects the adsorption energy. As the number of ions increases, the adsorption energy rises significantly.^[^ ^69^, ^80^ ^]^ This is attributed to the elevated electrostatic repulsive forces between the adsorbed ions or weaker interaction between the electrode surface and incoming ions. For example, when Li^+^ ions increased from 1 to 19 in Gr/C_2_N HR, the *E_AD_* increased from −3.034 to −0.121 eV.^[^ ^80^ ^]^ Therefore, *E_AD_* is an adequate parameter in determining the stability of any ion–electrode system, considering the influencing properties of ions and atoms.

M^+^ ion adsorption in HRs is also determined by the electronegativity difference between the electrode element and ion, the interlayer distance of bilayers, the number of ions, and the coordination number of ions with elements of the electrode.^[^ ^81^ ^]^ Consequently, the large electronegativity difference leads to a high binding affinity of ions with the host, dramatically increasing the adsorption energy. Wang et al. have investigated the effect of increasing BE in MoS_2_/WS_2_ HR on ions for LIBs.^[^ ^81^ ^]^ They observed that the increased BE of Li^+^ ion on the interface of MoS_2_/WS_2_ is higher due to the increased coordination number. The Li^+^ ion can bond with 6 S atoms in MoS_2_/Li/WS_2_, whereas it was bonded with only 4 S atoms in Li/MoS_2_ or Li/WS_2_. Therefore, HR configuration strongly binds the Li^+^ ions. Moreover, the effect of electronegativity can be understood by the combination of different MXene layers with BlackP.^[^ ^82^ ^]^ When V_2_CF_2_ and V_2_C(OH)_2_ are integrated with BlackP, they show different NAE (V_2_CF_2_: −2.4 eV, V_2_C(OH)_2_: −1.6 eV) due to nonidentical attached functional groups. V_2_CF_2_, because of the highly electronegative F atom, shows a high affinity toward alkali atoms compared to the ─OH group in V_2_C(OH)_2_. This manifests that V_2_CF_2_ is a better choice since it brings more stability due to strong ion interactions. Similar chemistry has been observed in other MXenes, such as Ti_3_C_2_F_2_/Ti_3_C_2_(OH)_2_, when assembled with BlackP for Na, Ti_2_CF_2_/Ti_2_CO_2_/Ti_2_C(OH)_2_ with Gr for Li, thereby confirming that the fluorine chemistries in HRs are advantageous for M^+^ ion storage.^[^ ^77^, ^83^ ^]^ Likewise, several HRs have been observed to attain higher NAE than their counterparts; the cumulative data from several reports is represented by a comparison bar plot in Figure 4b.^[^ ^43^, ^50^, ^55^, ^78^, ^84^, ^85^, ^86^, ^87^, ^88^, ^89^, ^90^, ^91^, ^92^ ^]^ Furthermore, such increased adsorption affinity proportionally improves the charge distribution of M^+^ ions over the interfaces of HR, indicating the elevated adsorption capacity of the material. The DOS and charge density difference of several HR, such as MoSe_2_/MXene, CoTe_2_/ZnTe, and MoS_2_/MXene, have been analyzed using theoretical calculations.^[^ ^62^, ^93^, ^94^ ^]^ The results depict that the densities around the interface of HRs are greater than those for individual layers. Overall, to boost ion‐electrode interactions and achieve higher capacities, optimizing *E_AD_* is strongly recommended.

### 3.3 Diffusion Energy Barrier

The *E_SD_* defines the energy that M^+^ ions require to migrate from one stable adsorption site to another through a metastable migration path inside the crystals of the anode material. The low *E_SD_* barrier with high mobility of ions within electrode materials is a key factor that significantly amplifies the rate capability of batteries.^[^ ^95^ ^]^ The energy barriers/activation energy for intercalating ions at different coordinate sites along various diffusion paths of the anode crystal structure are used to identify the mobility of ions. Among these, the diffusion path with the lowest energy barriers is more energetically favorable for the migration of ions. As mentioned above, there are four different sites (Figure 4a) for the adsorption of ions over the HR surface. After entering these sites, M^+^ ions further migrate from one coordination site to another (illustrated by a side view of HR in Figure 4c). There are several possible trajectories that ions can follow during migration. For example, if the ion has to migrate from atom 1 to atom 2 of layer 2 (Figure 4c, Top view), then there are three expected paths (path 1: through the atom of layer 1 located in the hollow site of layer 2, path 2: through the atom of layer 1 overlapped with an atom of layer 2, and path 3: through the bond between atoms). Ions will follow only that path which exhibits the lowest energy barrier. In the example of Ti_2_CS_2_/BlueP HR, path 1 and path 2 inevitably become path 3, which corresponds to the lowest *E_SD_* (0.37 eV) barrier. Notably, all types of M^+^ ions choose this path for their migration.^[^ ^43^ ^]^ Such results have been obtained for several HR, such as Sb_2_S_3_/SnS_2_,^[^ ^96^ ^]^ Ti_2_CS_2_/BlueP,^[^ ^43^ ^]^ Bph/Gr,^[^ ^50^, ^97^ ^]^ Gr/BlueP/MoS_2_,^[^ ^45^ ^]^ and so on. Moreover, it can also be observed that the *E_SD_* barrier for ions is lower when it sees the effect of two layers. The diffusion barrier of Li^+^ ions in the interlayer of Bph/Gr was significantly lower (0.618 eV) than that on the Bph surface (0.794 eV), indicating that Li^+^ ions can more easily diffuse in Bph/Gr.^[^ ^50^ ^]^ The decreased barrier for HRs has been widely observed, and many such reports are compared in Figure 4d.^[^ ^45^, ^50^, ^75^, ^85^, ^87^, ^88^, ^89^, ^90^, ^94^, ^98^, ^99^, ^100^, ^101^, ^102^, ^103^ ^]^ The stacked bar plot clearly expresses the decreased diffusion barrier of 2D HRs compared to the monolayers. Also, the energy barrier toward the diffusion of ions varies with coordination sites. Comparing the energy barriers for two paths (say 1 and 2), the values can be high at some locations and lower at other coordinate sites, as represented by the graphical plot in Figure 4e. Overall, the barrier for path 2 is the lowest and, hence, is the most favorable path for ionic diffusion.

Similar to *E_AD_*, the properties such as atomic size, electronegativity of each atom, their affinity with other layers, and the type of intercalating ions significantly alter the *E_SD_* barrier. For example, Wang et al. explained the influence of the high electronegativity of O atoms (3.61 eV) in Na_2_O than that of S (2.589 eV) and Se (2.424 eV) in Na_2_S and Na_2_Se, respectively. When the heterointerfaces between Na_2_O/Na_2_S and Na_2_O/Na_2_Se are formed, the O atom attracts surface Na atoms in the Na_2_S and Na_2_Se.^[^ ^104^ ^]^ This deviates the surface Na atom with the enlarged bond length between the Na atoms and the S/Se atom to exhibit localized atom aggregation areas near the Na_2_O surface, resulting in an increased diffusion barrier. Therefore, the Na_2_S/Na_2_Se HR with minimal electronegativity difference between S and Se showcases a low diffusion barrier. Furthermore, the impact of ionic properties on the diffusion barrier can be investigated using the example of VSe_2_/MoSe_2_ HR.^[^ ^101^ ^]^ The energy barriers are compared for Na^+^/K^+^ ion diffusion in the interlayer of VSe_2_/MoSe_2_ HR. It turns out that the *E_SD_* barriers for Na^+^ and K^+^ ions along hollow sites via saddle points are 0.21 and 0.11 eV, respectively. Both exhibit barriers lower than in the interlayer of MoSe_2_/MoSe_2_ homojunction (0.25 eV for Na and 0.13 eV for K). The larger mass and ionic size of K^+^ ions are expected to induce a higher diffusion barrier than Na^+^ ions. However, it is noteworthy that the diffusion of K^+^ ions experiences a lower barrier than Na^+^ ions. The orbitals of ions (Na s and K 4s) undergo hybridization with Se 4p orbitals, resulting in 3 Na─Se bonds (bond lengths: between 2.58 and 2.70 Å) and 2 K─Se bonds (bond lengths: 2.99 and 3.13 Å). This represents a strong interaction of Na─Se compared to K─Se, which leads to a decreased diffusion barrier or fast kinetics for K^+^ ions.^[^ ^101^ ^]^ A similar trend was observed for the diffusion of Li^+^, Na^+^, and K^+^ ions in preloaded VS_2_/MXene HR by M^+^ ions.^[^ ^46^ ^]^ The preloaded M^+^ ions on the surfaces of VS_2_ and MXene offer barriers to the migration of ions. The existence of guest ions in the path increases the barrier at such locations. For instance, the barrier rose from 0.2 to 0.48 eV on the VSe_2_ surface and 0.2 to 0.59 eV over the MXene surface for Li^+^ ions, whereas these increased to more than 0.6 eV for Na^+^ and K^+^ ions. The higher barrier for Na^+^ and K^+^ ions accounts for their larger ionic sizes. However, an opposite trend was observed when the migration of ions was analyzed for the preloaded surfaces of VS_2_ and MXene by double layers of Li/Na/K metals. The barrier for all ions decreased over double M^+^ ion layer‐coated TMDs and MXenes (0.06, 0.03, and ≈0.014 eV for Li^+^, Na^+^, and K^+^ ions, respectively). The lower barriers for Na^+^ and K^+^ than Li^+^ ions are due to the weak adhesions or lower BEs of these ions with the TMD surface due to their larger sizes, leading to their fast mobility. The findings also demonstrated that the diffusivities and ionic conductivities (*σ*) of Li^+^ ions at 40% and 80% concentrations are higher than those of the 100% cases, whereas, for Na^+^ and K^+^ ions, these always increase with loading concentration.^[^ ^46^ ^]^ Again, in Ti_2_CS_2_/BlueP HR, the *E_SD_* barriers for Li^+^, Na^+^, and K^+^ ions are 0.37, 0.21, and 0.10 eV, respectively, due to the abovementioned reasons.^[^ ^43^ ^]^ Therefore, the *E_SD_* barrier is a fundamental parameter that can be adopted to understand the diffusion mechanism in different materials. This factor highly influences the migration of M^+^ ions, and its optimization for different 2D HRs can help in developing faster batteries.

### 3.4 Lattice Deformation

The stability of the lattice structure of electrode material during the multistep diffusion process of M^+^ ions is crucial for long‐term performance. The structural stability is highly influenced by certain factors such as interplanar spacing and mechanical index of electrode material, ionic radii of M^+^ ions, the type of bond (ionic, covalent, or vdW), and the bond length between M^+^ ions and distinct atoms belonging to layers of HR.^[^ ^105^ ^]^ Continuous charging–discharging of M^+^ ions, specifically Na^+^ and K^+^ ions, simultaneously drives expansion‐contraction of the crystal structure, respectively. Such changes induce pulverization (when the material gets fragmented and changes its original structure) and volume expansion, completely destroying the structure within a few cycles, as shown in Figure 4f. This issue is mainly prominent in anode materials, where volume expansion can reach 300% for alloying‐type materials such as SnS_2_ and nearly 100% for conversion‐based materials such as Fe_2_O_3_, compared to cathodes, where expansion is negligible (<25%).^[^ ^106^ ^]^ Unfortunately, continuous pulverization of electrode material can break the SEI layer, which further brings about the issue of ion loss and irreversible capacity. In addition, during the alloying and conversion reactions, the electrode material undergoes several phase changes that result in the agglomeration of poorly conducting inactive species. These changes bring cracks in the electrode materials, and eventually, the capacity of the device fades within a few cycles, paving the way to the early death of the battery. Such deformations are more likely to happen at higher current rates. It becomes imperative to identify the mechanical stability of electrode materials in order to design stable batteries. Several computational and experimental techniques have been employed to investigate the construction of 2D HR, aiming to mitigate the effects of pulverization and expansion. Recently, in an investigation, researchers observed that the restacking and agglomeration of SnS_2_ can be suppressed by creating a rigid interface with MoS_2_, thereby improving its mechanical strength. The expansion ratio for SnS_2_/MoS_2_ was calculated to be 2.45% only, corresponding to 3.27–3.35 Å interlayer change, which is much lower than that for SnS_2_ (7.26%), and MoS_2_ (4.64%) monolayers.^[^ ^74^ ^]^ Therefore, the construction of 2D HRs is a viable approach to restrain the volume changes in anode materials. In this regard, the stiffness of these electrode materials against the strain produced by diffusing ions must be sufficiently high to reduce the deformation. The in‐plane stiffness constants (*C_ij_*) of HRs when lattice changes along “*i*” and “*j*” directions can be evaluated using Equation (8), and the corresponding tensile strain (*ε*) by Equation (9), which are given as:^[^ ^107^ ^]^

(8) $$\begin{array}{c} \;C_{ij}=\frac{1}{A_{0}}\;\frac{\partial^{2}E_{s}}{\partial \varepsilon_{i}\varepsilon_{j}}\; \end{array}$$

(9) $$\begin{array}{c} \varepsilon =\frac{\left(a-a_{o}\right)}{a_{o}} \end{array}$$

where *E_s_* is the total elastic energy per unit cell, *a* represents the value of the lattice constant corresponding to strained HR, *a_o_* is the original lattice constant, and *A*_0_ denotes the equilibrium surface area of the supercell. For Gr/BlueP/MoS_2_ HR, the value of stiffness constant (*C*_11_) is much higher (600.33 N m^−1^) than those obtained for Gr (340 N m^−1^), MoS_2_ (120 N m^−1^), and BlueP (80 N m^−1^), indicating upgraded mechanical stability of HR.^[^ ^45^ ^]^

Using Equation (8), the Young's modulus (*Y*) and Poisson's ratio (*ϑ*) for 2D HRs can be evaluated as:^[^ ^107^, ^108^ ^]^

(10) $$\begin{array}{c} \;Y_{xx\left(yy\right)}=\frac{{C_{11\left(22\right)}}^{2}-\;{C_{12}}^{2}}{C_{11\left(22\right)}} \end{array}$$

(11) $$\begin{array}{c} \;\vartheta_{xx\left(yy\right)}=\frac{C_{12}}{C_{11\left(22\right)}} \end{array}$$

provided that they satisfy Born–Huang criteria (*C*_66_ > 0 and *C*_11_ *C*_22_ − *C*_12_^2^ > 0) for mechanical stability. The in‐plane *Y* values [from Equation (10)] describe the ability of the HRs to resist deformation created by the intercalating/de‐intercalating ions, while *ϑ *values [from Equation (11)] reflect the extent of deformation observed in different directions during the charge–discharge process. For prolonged cycle stability, HR materials must possess high strain capability or elevated values of *Y* coupled with low *ϑ *values. Unfortunately, most redox‐active 2D materials, mainly belonging to families of TMDs and TMOs, exhibit poor mechanical strength and flexibility, restricting their effectiveness in maintaining stable device performance. Integrating these materials with flexible 2D materials like Gr, MXenes, P, and Bph can significantly enhance their stability. As an example, *Y* values for MoS_2_/Ti_2_CO_2_ and MoS_2_/Ti_2_CF_2_ HR are 370.6 and 309.6 N m^−1^, respectively, which are much higher than that of their monolayer counterparts (MoS_2_: 139.2, Ti_2_CF_2_: 178.9, and Ti_2_CO_2_: 213.2 N m^−1^), outlining the improved mechanical strength of MoS_2_ when combined with MXene.^[^ ^78^ ^]^ Also, the *ϑ * for MoS_2_/Ti_2_CO_2_ and MoS_2_/Ti_2_CF_2_ reduced to 0.274 and 0.265 than those of monolayered Ti_2_CO_2_ (0.299) and Ti_2_CF_2_ (0.308), respectively, signifying less deformation of HR.^[^ ^78^ ^]^ Ma et al. also demonstrated the improved mechanical strength of VS_2_ by hybridizing it with different MXene (Ti_3_N_2_T_2_; T = F, O, OH) layers.^[^ ^109^ ^]^ The results indicated that the Ti_3_N_2_O_2_/VS_2_ attained highest Y (463.14 N m^−1^) value compared to Ti_3_N_2_F_2_/VS_2_ (361.36 N m^−1^) and Ti_3_N_2_(OH)_2_/VS_2_ (338.47 N m^−1^), which aligned with strength of corresponding monolayers (Ti_3_N_2_O_2_: 344.95, Ti_3_N_2_F_2_: 232.92, Ti_3_N_2_(OH)_2_: 238.58, and VS_2_: 107.88 N m^−1^). Thus, selecting appropriate monolayers is necessary to improve mechanical stability in their HR. Similarly, *Y* of VS_2_/Gr (434.9 N m^−1^) is higher than that of Gr (340.6 N m^−1^) and VS_2_ (87.5 N m^−1^) monolayer; for BlueP/MoS_2_ (203.58 N m^−1^) it is higher than MoS_2_ (127.038 N m^−1^) and BlueP (37.14 N m^−1^).^[^ ^102^, ^110^, ^111^, ^112^ ^]^ The enhanced values of *Y* reflect the strengthened structural deformation resistance toward intercalating/de‐intercalating ions in anodes, further highlighting the synergistic effect of two monolayers in designing stable electrodes.^[^ ^102^ ^]^

The issue of pulverization is mainly associated with the larger‐sized ions (Na^+^ and K^+^), which find it difficult to diffuse through crystalline structures, and their sluggish kinetics destroy the structures.^[^ ^113^, ^114^ ^]^ Regulating the crystallinity of these HR materials by introducing defects can further bring flexibility that minimizes pulverization and lattice expansion during diffusion. Ma et al. have excellently outlined the boosted stability of amorphous MoS_3_/rGO HR compared to crystalline MoS_2_/rGO HR for SIBs and PIBs.^[^ ^115^ ^]^ Using Raman spectra, they calculated the strain introduced (*δ*) due to amorphous features by the equation:^[^ ^115^ ^]^

(12) $$\begin{array}{c} \delta =2.66\;\frac{\Delta \omega }{\omega } \end{array}$$

where *ω* is the wavenumber and *∆ω* is the change in wavenumber. The blueshift of 2 cm^−1^ in MoS_3_/rGO compared to MoS_3_ indicated a strain of 1.9%. Such strain mitigates the volume expansion; the MoS_3_/rGO exhibited only a 15.8% expansion ratio during the sodiation process, significantly lower than the 61.1% observed for MoS_2_/rGO. The Na^+^ ions require substantial energy to expand the (002) layers of MoS_2_/rGO HR, and the continuous charge–discharge process led to fragmentation and the disappearance of the (002) peak. In contrast, the internal free volume in amorphous MoS_3_/rGO relieved the sodiation strain and pulverization, helping to retain stability till 40000 cycles.^[^ ^115^ ^]^

Furthermore, for the flexible ESDs, the materials should be able to accommodate larger strain at a specified external stress, i.e., their *Y* values should be low. Gr, owing to excellent mechanical strength and flexibility (*Y* ≈ 340 N m^−1^), is considered an optimal candidate for flexible batteries, as it is less prone to pulverization. Several HRs with *Y* values comparable to or lower than that of Gr have been designed to diversify this. For instance, HR materials such as BlueP/NbS_2_ (102.65 N m^−1^),^[^ ^23^ ^]^ BlueP/SMoSe (195.9 N m^−1^),^[^ ^116^ ^]^ and AlN/VS (249.69 N m^−1^),^[^ ^117^ ^]^ exhibit much lower *Y* values and, consequently, higher flexibility. Moreover, the *Y* for lithiated BlueP/SMoSe (accommodating 24 Li atoms) further reduced to 138.48 N m^−1^, indicating its high flexibility to confront expansion due to the large number of intercalating ions.^[^ ^116^ ^]^ Thus, optimizing the value of *Y* based on the specific requirements for flexibility and rigidity in batteries tailored to their intended applications is essential.

Interestingly, the bandgap of HRs can be reduced by applying the reversible strain.^[^ ^98^ ^]^ Such strain engineering substantially improves the conductivity of HR, which further enhances its adsorption properties. The DOS plots can express the increased electronic density near the Fermi level due to the application of strain, as observed for BlueP/MoS_2_,^[^ ^112^ ^]^ and WO_2_/WS_2_,^[^ ^98^ ^]^ HR, where the material transitioned from the semiconducting to the metallic phase. Such a strategy benefits poorly conducting electrode materials belonging to TMDs, TMOs, and alloys. Therefore, enhancing the mechanical properties of layered structures is extremely supportive to avoid the degradation in cycling performance caused due to expansion/pulverization, making the HR materials suitable as an electrode material for MIBs.

Overall discussions suggest that a selective combination of monolayers can provide the 2D HRs with commendable adsorption and diffusion properties, enhanced mechanical strength, ionic/electronic conductivity, and stability. The comprehensive details of each fundamental feature of 2D HRs associated with respective controllable parameters, observations, and contributions to understanding the performance of batteries are presented in **Table** **2**.^[^ ^23^, ^43^, ^74^, ^77^, ^79^, ^97^, ^112^, ^118^, ^119^, ^120^, ^121^, ^122^, ^123^ ^]^

**Table 2:** The summary of different theoretical investigations performed using DFT calculations in battery applications. It also highlights some of the controllable parameters for each method, corresponding observations, and their contributions toward understanding the key characteristics of batteries.

Technique	Analysis method	Parameters	Observations	Contribution toward battery performance	Refs.
Theoretical investigations					
DFT calculations (Software: Vienna ab initio simulation package, Quantum espresso)^[^ ^94^ ^]^	Density of states (DOS)	Exchange‐correlation functional, pseudopotentials and basis set, K‐point sampling, convergence criteria, charge and spin state, supercell size and periodic boundary conditions, lithium/vacancy diffusion barriers, electrolyte environment, temperature and finite‐temperature effects, thermodynamic stability and free energy fluctuations, smearing and occupation	Electronic band structure of system, Fermi level, electron migration	Materials with controlled conductivity, structural properties, doping concentrations, and redox activity can be designed. Predicts reaction voltage by Gibbs free energy, the evolution of SEI and CEI layers, side products formation, etc.	[118]
	Projected density of states (PDOS)		Contribution from specific atoms or orbitals in electronic band structure	Determine the role of each element in the battery mechanism by analyzing changes in oxidation states and electronic states. The effects of doping, surface modifications, ion size, etc., can be predicted.	[43]
	Bader charge analysis		Charge transfer between M^+^ ions and atoms of HR	The conductivity of electrode materials can be optimized by selecting appropriate combinations.	[119, 120]
	Adsorption energy		The energy required by M^+^ ions to adsorb at different sites of electrode material surface.	Specific capacity, energy density, and rate capability of batteries can be optimized.	[77, 79]
	Binding energy		The interaction energy between layers of HRs and with M^+^ ions.	Rate capability optimization, determines favorable conditions for the ion diffusion.	[121, 122]
	Diffusion energy barrier		Diffusion energy barrier	Energetically favorable diffusion path of ions in the crystal structure can be known. It helps find appropriate crystal structures promoting ion migration.	[74, 97]
	Strain expansion		Stiffness coefficients, Young's modulus, bulk modulus, lattice expansion.	Issues such as pulverization, particle size change, clustering, and lattice deformations can be analyzed.	[23, 112, 123]

## 4 Synthesis Approaches for 2D HR

**Figure 5:**
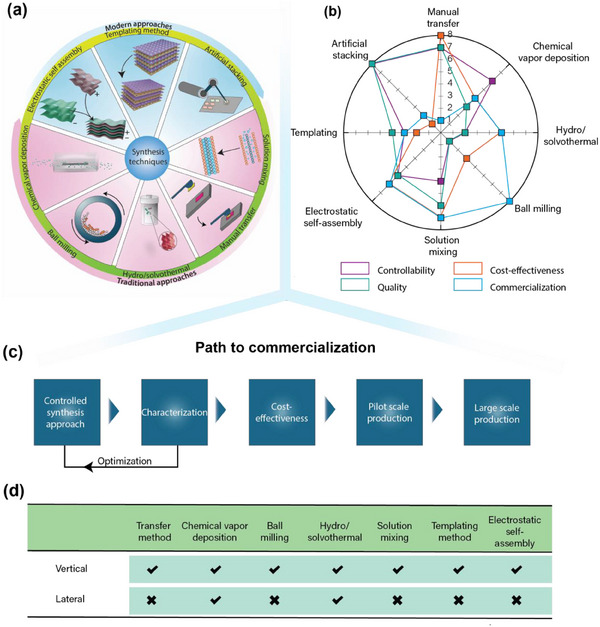
a) The diagram illustrates various synthesis methods categorized under modern and conventional synthesis approaches. b) The radar plot displays the selective comparison of different synthesis methods from the commercialization viewpoint.^[^ ^131^, ^189^ ^]^ The highlighted factors include mass production, crystal quality, cost efficiency, and layer‐by‐layer controllability for 2D HR electrode materials. c) The flowchart representing the major steps followed by industry to optimize battery device for large‐scale production. d) An overview of the suitable methods for synthesizing vertical and lateral HRs (transfer method includes manual and artificial stacking).

2D HRs have been utilized in a broad range of applications. For the effective contribution of each layered structure, it is preferable to employ a layer‐by‐layer architecture with a maximum overlapping area instead of a few layered structures. However, precisely controlled fabrication of such HR materials, particularly for covalently bonded lateral HR, is challenging. Identifying a method that effectively facilitates layer‐by‐layer assembly at a large scale without added impurities is necessary. Many techniques are introduced for synthesizing 2D HRs, each with its own strengths and weaknesses, and can be categorized as conventional and modern approaches (**Figure** **5**a).

### 4.1 Conventional Methods

#### 4.1.1 Manual Transfer Method

This method has gained widespread application in microelectronics. It involves manually assembling the 2D layers over a target substrate following the “pick‐and‐transfer” approach, as shown in Figure 5a. Depending on the medium used to transfer these layers on the target substrate, this method is further categorized as a wet transfer (aqueous medium) and dry transfer (using glue‐type material or tape) approach.^[^ ^124^, ^125^ ^]^ For instance, for wet transfer of Gr grown on Cu foil, a polymer (e.g., PMMA, PVA) supporting layer is spin‐coated on it, and then the Cu substrate is etched away by the corresponding etchant. Subsequently, the polymer/Gr stack is picked up by the target substrate, such as Si/SiO_2,_ and the polymer layer is dissolved in organic solvents.^[^ ^126^ ^]^ However, in the dry transfer method, the elastomer stamp, such as PDMS, is used to pick up the 2D layers from their original substrate and release them on the target substrate based on the principles of viscoelastic stamping (pressing and heating).^[^ ^127^ ^]^ Through repetitive layering of 2D layers onto the target substrate, high‐quality micrometer‐sized 2D HRs with robust ionic, covalent, or vdW interactions are produced. 2D layers of appropriate sizes can be selected to obtain completely overlapped layer‐by‐layer vertical HR assemblies. However, this method lacks the atomic precision required for the realization of chemically bonded lateral HRs. The laterally placed layers using this method can have physical interaction, but do not facilitate the formation of heterojunction. Also, the vertically aligned HRs can contain microlevel impurities, such as polymers or solvent bubbles over the surfaces, which disrupt their electronic conductivity.^[^ ^128^ ^]^ In addition, preparing a stack of four layers takes a lot of human power and time. Consequently, these disadvantages of this technique do not permit its scalability in quantity and time, limiting its application in large‐scale battery production.

#### 4.1.2 Vapor Deposition

The chemical vapor deposition (CVD) method is another approach that extensively synthesizes millimeter‐sized 2D HRs for various applications. This technique generates vapors from solvent or powder materials under optimized temperature and pressure conditions and is further directed toward the target substrate through a gas flow. This leads to the nucleation of vapors over the substrate to form a monolayer.^[^ ^129^ ^]^ The size, structure, shape, thickness, and properties of layers are intricately dependent on external controllable conditions, such as temperature, substrate positioning, precursors, reaction rate, and carrier gas atmospheres. This allows tuning of HRs to obtain their desired properties. By implementing a multistep CVD process, sequential layer growth yields high‐quality HRs with minimal defects and impurities.^[^ ^130^ ^]^ Moreover, using this technique, the transfer step can be skipped, and there is direct growth of meticulously controlled vertical or lateral 2D HRs on the substrates. However, this technique is preferentially used to grow HRs of monolayers with similar crystal structures, specifically TMDs and TMOs.^[^ ^131^, ^132^ ^]^ Such millimeter‐sized 2D HRs with constraints of specified crystal structure features can be explored in microbatteries, but are not beneficial for large‐scale battery fabrication. Notably, this technique has been widely employed for batteries to design HRs directly over current collectors as the substrate. As an example, Yu et al. deposited Sb_2_Te_3_ sheets directly over the Cu foil substrate using the CVD method and then spin‐coated the carbon layer over its surface.^[^ ^133^ ^]^ The foil was cut into circular discs to use as the electrode for SIBs, and such a strategy helped in attaining stable performance even after 1000 cycles at 2 A g^−1^ current density. In another approach for VSe_2_/Gr(V) HR, firstly, Gr is vertically (V) grown over carbon cloth (CC) substrate by plasma‐enhanced CVD incorporated with CH_4_ gas.^[^ ^134^ ^]^ After that, Se and vanadium chloride precursors were used to grow VSe_2_ over Gr@CC via an atmospheric pressure CVD. Similarly, multistep CVD can help in obtaining HRs with different chemical compositions. Beyond this, it is well known that the high conductivity of electrode materials favors the mobility of electrons and ions to give high‐rate performance in batteries. Several carbon‐sourced solvents, such as acetonitrile, ethanol, benzene, toluene, and so on, are also utilized for carbon layer deposition over different substrates or HR surfaces using vapor deposition to obtain the conductive surface.^[^ ^74^, ^135^ ^]^

Atomic layer deposition (ALD) is another vapor deposition method for obtaining thin films (<10 nm) through layer‐by‐layer coating of the precursor gas vapors onto substrates. In this method, the precursors react in a self‐restricting manner, and the reaction stops when precursor gases react with the overall substrate sites.^[^ ^136^ ^]^ Uniform layered HRs with atomic‐level precision thickness are realized and capable of depositing HRs of larger size than those typically obtained in CVD.^[^ ^137^ ^]^ This method is utilized to design several anode materials by directly depositing active materials over current collectors or manipulating their surfaces to enhance and regulate battery performance.^[^ ^138^, ^139^ ^]^ This includes reduced volume expansion, increased conductivity, and restricted dendrite growth.^[^ ^138^, ^139^ ^]^ Despite these advantages, ALD is a highly time‐consuming and expensive technique with low‐volume 2D HR active materials production.^[^ ^140^ ^]^ Overall, both of these methods use a large amount of energy and time to give millimeter‐sized HR assemblies. While such HR can be incorporated in micro/nanobatteries in the market, it is not applicable to large‐scale manufacturing. Therefore, exploring alternative approaches for quantitative and qualitative material synthesis has become essential.

#### 4.1.3 Solution Mixing

The solution mixing or wet assembly method involves the synthesis of HRs in a liquid‐based medium under specified conditions to regulate their growth. This is a facile and low‐cost method that provides a high yield of synthesized materials. The strategies followed are generally tailored depending on the type of composition of individual layers. For already existing 2D structures, ionic, covalent, or vdW interactions, or other external forces (provided by sonication or stirring) infuse different layers between one another.^[^ ^141^ ^]^ In the most followed pattern, the precursors of exfoliated layers with different compositions are dispersed in a solvent, and smaller 2D sheets like MoS_2_ and SnS_2_ get diffused inside the large‐sized layered materials such as GO and MXenes.^[^ ^47^ ^]^ The high aspect ratio of large layered materials facilitates the rapid diffusion of small layers and further provides stability to the final HR.^[^ ^142^ ^]^ The assembled HRs can be further retrieved by filtration, freeze drying, or centrifugation.^[^ ^143^, ^144^ ^]^ In a recent study, the reliability of this method is highlighted by the controlled synthesis of the BlackP/MXene HR for SIBs. The researchers fabricated the BlackP/MXene by thoroughly mixing the few‐layered colloidal solutions of BlackP and MXene and allowing their self‐assembly through a vacuum freeze‐drying process.^[^ ^77^ ^]^ The robust structure of BlackP/MXene prevented the pulverization of BlackP due to the conductivity and flexibility of MXene, and it retained 467 mAh g^−1^ capacity after 100 cycles compared to that for a mechanically mixed random BlackP‐MXene mixture (171 mAh g^−1^).

In a different modified approach, layers of desired material are firstly dispersed in a solvent, followed by in situ growth of another layer over the former.^[^ ^145^, ^146^, ^147^ ^]^ This facilitates the layer‐by‐layer growth of HRs, mitigating the restacking issues associated with the 2D materials. Following this strategy, Chen et al. successfully infused MoS_2_ between the MXene layers (Mo_2_TiC_2_T_x_). They first introduced S particles in the layers of exfoliated Mo_2_TiC_2_T_x_ and further treated it at higher temperatures (>500 °C) to achieve MoS_2_/MXene HR.^[^ ^147^ ^]^ MoS_2_/Mo_2_TiC_2_T*_x_* HR delivered 2.4 times greater capacity than pure Mo_2_TiC_2_T*_x_*. The enhanced performance is attributed to the open structure of MoS_2_/Mo_2_TiC_2_T*_x_*, which offers a smaller diffusion resistance than that of the restacked Mo_2_TiC_2_T*_x_* sample. Following a similar approach, WS_2_, as well as MoS_2_/WS_2_ HR, can be infused between MXene layers.^[^ ^145^ ^]^ The growth of the structure can be controlled by temperature, which can further prevent the re‐stacking of the same layered materials. Similarly, Wang and his coworkers synthesized 2D‐2D MXene@Co_9_S_8_/CoMo_2_S_4_ by first growing uniformly tightly packed ZIF‐67 over MXene in a methanol solution.^[^ ^148^ ^]^ This is followed by the ion exchange of Co^2+^ by selectively etching MoO_4_^2−^ in the precursor to obtain the 2D‐2D MXene@CoMo‐LDH intermediate. Finally, the in situ sulfidation process leads to the formation of highly flexible and conducting MXene@Co‐Mo‐S HR, giving much better retention (≈65%) than the Co‐Mo‐S sample (≈30%) for Na^+^ ion storage. MXene layers in Co‐Mo‐S help minimize agglomeration during the continuous charge–discharge process and, hence, help maintain stable performance.^[^ ^148^ ^]^ In another approach, the in situ epitaxial growth of graphdiyne (GDY) is obtained in the MXenes lamellae using HEB (hexaethynylbenzene) monomers in *N*, *N*‐dimethylformamide solvent for 3 days.^[^ ^149^ ^]^ The controlled growth expanded the interlayers of MXene from 0.27 to 0.34 nm and tackled the issue of restacking. The expanded layers provide easy access to the large‐sized Na^+^ ions (0.106 nm).^[^ ^149^ ^]^ Overall, several HRs with variable configurations and properties can be optimized using this synthesis route. Since there is no requirement for sophisticated instruments, this method is highly favorable in synthesizing materials for batteries and other large‐scale energy storage applications. However, this method has a probability of solvent impurities, unreacted precursors, and nonuniform and uncontrolled growth of HR. Also, this method is favorable only for vertical 2D HR.

#### 4.1.4 Hydrothermal/Solvothermal Method

Hydrothermal (aqueous solvents)/solvothermal (nonaqueous solvents) is the facile, scalable, and low‐cost solution‐based route to synthesize the layered and 2D HRs under low temperature (120–250 °C) and high pressure (≈10^5^ Pa) conditions. This method helps in achieving distinct phases of samples by their sequential nucleation. Generally, the strategy followed to synthesize 2D HRs includes mixing one or more 2D nanosheet materials with the precursors of other materials.^[^ ^150^, ^151^ ^]^ The large‐sized layer, such as GO or MXenes, provides nucleation sites to grow later material with a layered structure.^[^ ^152^, ^153^, ^154^ ^]^ The mixed solution is then transferred to autoclave reactors under ambient conditions. For example, Dai et al. utilized this method to synthesize 2D MXene/V_2_O_5_ HR, where exfoliated MXene sites were used to grow V_2_O_5_ sheets.^[^ ^155^ ^]^ The obtained gel was further freeze‐dried and pressed along a specific direction to obtain vertically (V‐MXene/V_2_O_5_) and horizontally (H‐MXene/V_2_O_5_) aligned 2D HRs. V‐MXene/V_2_O_5_ HR electrode contains vertically aligned channels in a “sheet‐on‐sheet” manner, which allows fast electron/ion mobility, minimizes the volume expansion, and avoids the use of inactive agents (metal current collectors, conductive additives, and polymer binder). Such characteristics facilitate the reversible M^+^ ion diffusion and improve capacity (463 mAh g^−1^ at 0.2 A g^−1^) and rate capability compared to H‐MXene/V_2_O_5_. Impressively, Fan et al. synthesized VS_2_/MoS_2_ HR using the hydrothermal method and outlined its advantage over the mechanically prepared VS_2_‐MoS_2_ mixture.^[^ ^156^ ^]^ The results demonstrated that due to heterojunction formation in VS_2_/MoS_2_ HR, the material could maintain a capacity of 714.2 mAh g^−1^ at a high rate of 2 A g^−1^, which is superior to the performance of the VS_2_‐MoS_2_ mixture (330.6 mAh g^−1^). The better charge capability of VS_2_/MoS_2_ than the VS_2_‐MoS_2_ mixture prevents it from “under‐voltage failure” caused by dendrite formation at higher current rates.

For batteries, a stable electrode material allowing reversible movement of ions is imperative. Therefore, the heterojunction between different layers of HRs should be strong enough to accommodate volume changes occurring during the intercalation/de‐intercalation of ions. The hydrothermal method allows interface‐controlled crystallization of 2D HRs. As an example, the interface coupled SnSe_2_/Gr HR is synthesized by simply mixing precursors and treated at 180 °C in an autoclave.^[^ ^57^ ^]^ The strong coupling of SnSe_2_ with Gr sheets buffers the volume change and reduces the agglomeration of Sn generated during alloying‐conversion reactions, and helps in attaining long‐term performance for 1500 cycles at 0.5 C.

This route has always remained successful for the synthesis of vertical as well as lateral HRs, including a variety of compositions.^[^ ^150^, ^157^, ^158^ ^]^ To obtain SnS_2_/Sn_0.5_Mo_0.5_S_2_ lateral HR, Wang et al. followed a two‐step hydrothermal process. Firstly, hydrothermal was employed to prepare SnS_2_ sheets, and further precursors of Mo and S were added together with SnS_2_ to finally obtain SnS_2_/Sn_0.5_Mo_0.5_S_2_ lateral HR. It is noteworthy that no expensive facilities such as CVD and ALD are required, and a large amount (in grams) of material can be synthesized using this technique. This is the most suitable method for scalable production with controlled compositions. However, the synthesized HRs have high possibilities of agglomerated particles as well as unclean surfaces of 2D layers. Also, it is impossible to control the number of layers and direction of growth using this technique.

#### 4.1.5 Ball‐Milling

This technique is attributed to the shear force induced by the moving balls to create a few‐layered 2D HRs. It is the easiest method to exfoliate the bulk materials into a few layered materials and further infuse the different layers into each other with the impact of high energy. Moreover, the obtained structure depends on the force or energy produced by agate balls, their rotation speed, density, and size. Depending on the media, this method is further instigated as wet ball milling (WBM) and dry ball milling (DBM). WBM is done in the presence of some solvent (N‐methyl‐2‐pyrrolidone [NMP], dimethylformamide [DMF], and de‐ionized water) acting as a lubricant as well as energy transfer media to exfoliate the layers, whereas DBM is done in the presence of some agents (NaCl, dry ice, melamine) which are added to reduce the effective force on the main sample.^[^ ^159^, ^160^ ^]^ However, after the milling process, these additives need to be removed by some chemical treatments to purify the active materials. WBM is a more appreciable method than DBM due to the low cost (no cost of additives), fewer possible impurities, and high efficiency.^[^ ^79^, ^161^, ^162^, ^163^ ^]^ Since bulky layered materials are directly exfoliated, and HRs can be assembled easily, this technique is very prominent for mass production and optimization of ratios of different layers. For its cost‐effectiveness, wet ball milling utilizing water should be favorable instead of expensive solvents. Besides, it is to be highlighted that this technique is suitable only for a few‐layered HRs and is not dependent on the lattice parameters.^[^ ^161^ ^]^

### 4.2 Modern Approaches

#### 4.2.1 Electrostatic Self‐Assembly

Electrostatic self‐assembly is a solution‐based method where the 2D layers from different materials are first made to gain opposite electrostatic charges individually. Furthermore, both are mixed, and the oppositely charged surface induces the electrostatic attractive force, which results in the self‐assembly of layers over each other. In 2017, Gogotsi et al. synthesized flexible 2D MXene/rGO HR with negative and positive charges over MXene and rGO, respectively.^[^ ^164^ ^]^ The 2D rGO layers were made positive by dispersing in poly(diallyl dimethylammonium chloride) (PDDA) polymer, whereas MXene (Ti_3_AlC_2_) inherits the negative charge due to attached functionalities. Freestanding HR films were formed after mixing the solutions and resting them for some time. MXene and Gr‐based several other HRs have been realized using this method for batteries since their surface can be functionalized easily.^[^ ^70^, ^165^, ^166^ ^]^ The polymers containing ionizable species in their chains, such as PDDA, polyaniline, tris(2‐aminoethyl) amine, poly(ethylenimine), poly(allylamine hydrochloride), and cetyltrimethylammonium bromide (CTAB), containing an amine group, are widely used for providing positively charged species.^[^ ^165^, ^167^, ^168^ ^]^ Moreover, compounds such as sodium lauryl sulfate, poly(styrenesulfonic acid), poly(vinyl sulfonate), poly(acrylic acid), and poly(methacrylic acid) containing anionic groups such as ─COO^−^, ─SO_3_^−^, ─OSO_3_^−^, and ─OPO_3_^−^ have been incorporated to develop negative charges.^[^ ^169^ ^]^ The well‐known zeta potential analyzer measures the charge acquired by different layered structures. To evaluate the efficacy of this method, Sasaki et al. conducted a comparative analysis using conventional random self‐assembly and electrostatic self‐assembly methods for fabricating MnO_2_/Gr and MoS_2_/Gr 2D HR.^[^ ^170^, ^171^ ^]^ The results demonstrated that electrostatic self‐assembly facilitated layer‐by‐layer stacking of HRs in contrast to randomly mixed HR, which consisted of a few layered structures prone to restacking. Therefore, this method presents a robust way to achieve ordered layer‐by‐layer vertically stacked 2D HR. Despite these promising achievements, the method is still in its preliminary period and encounters significant challenges, particularly in controlling charge distribution. The difficulty in precisely managing the charges/functionalities solely across the edges of layers limits its applicability for realizing lateral HRs. Moreover, this method needs to be extended to hybridize other layered materials beyond MXene and Gr. Also, the high cost associated with the polymers used to charge the materials demands some cost‐effective alternatives. Cheaper pathways to generate charges, such as utilizing simple bases (NaOH, KOH, etc.), following electrochemical routes, and targeting inherited charged properties of different 2D materials, need to be explored. Overall, this synthesis technique can be welcomed as a great way to assemble 2D vertical HRs easily.

#### 4.2.2 Templating Method

The surface manipulation of monolayered structures by introducing porosity tremendously increases the active sites by turning them from impermeable to sieving‐type structures, allowing fast mobility of ions. These tuned nanoarchitectures promote their better reaction kinetics even for large‐sized K^+^ ions. Nonetheless, the confined 2D nanoporous HRs integrating the uniform holey layers of different compositions are challenging to realize by limited methodologies. Various methods, particularly chemical and soft/hard templating, have been developed to construct 2D porous architectures in HRs.^[^ ^103^, ^172^, ^173^, ^174^ ^]^ In the hard templating approach, MgO, SiO_2_, metal–organic frameworks (MOF), and so on, templates are used to direct the growth of 2D materials and are further etched out to leave behind porous architecture.^[^ ^175^ ^]^ The etching process required toxic chemicals, making this method environmentally unfriendly. By contrast, the soft‐templating method is the most prominent approach, allowing the creation of porous layers with controlled pore sizes and shapes. This method utilizes micelles of block copolymers (BCPs) and surfactants, which are removed by temperature treatments to create controlled porous layers.^[^ ^176^, ^177^ ^]^ For example, soft templates such as F127 and P123 are mixed with a carbon source (resol or dopamine) and then further added to other 2D layered material‐carrying solutions.^[^ ^178^, ^179^, ^180^, ^181^ ^]^ This facilitates the infusion of micelles carrying carbon sources between bilayers of 2D structures. Afterward, under an inert environment at preoptimized temperature conditions, the carbonization is done along with the removal of BCPs, leaving behind the porous carbon layers between other 2D bilayers. For instance, Zhao et al. fabricated unique mesoporous C/TiO_2_/C HR using F127 micelles.^[^ ^178^ ^]^ The highly conductive and mesoporous structure promoted the reversible movement of Na^+^ ions and aided in attaining outstanding performance (73 mAh g^−1^ specific capacity) at a high rate of 20 A g^−1^. Also, the reversible channels for ions and pseudocapacitor behavior of HR allow it to attain 77.8% stability after 20000 cycles at 10 A g^−1^. In a similar approach, 2D mesoporous carbon/MoS_2_ HR has been generated by self‐assembly of F127‐resol micelles over exfoliated 2D MoS_2_ sheets.^[^ ^182^ ^]^ The heterointerfaces and porous structural features allow fast transportation of electrons and Li^+^ ions even at higher rates (400 mAh g^−1^ at 10 A g^−1^). In addition, the HR exhibits almost 2.5 times higher performance than the MoS_2_, attributed to the increased conductivity due to coupled interfaces with carbon. Similarly, Wang et al. synthesized sandwiched C@MXene@C structure using F127 BCP and melamine‐formaldehyde (MF) resin as a source of carbon.^[^ ^183^ ^]^ Slow evaporation of the solvent led to the assembly of F127/MF micelles over Ti_3_C_2_T_x_ nanosheets. The synthesized composite is then further carbonized and loaded with S to use as anode material for batteries. Following this strategy, various combinations of porous 2D HRs are possible using TMDs, TMOs, MXene, and Gr, with mesoporous carbon.^[^ ^184^, ^185^ ^]^ This method can provide fascinating materials for energy storage applications. However, the low material yield along with the high cost of templates are the limiting factors for the commercial application of this method. Consequently, it is necessary to develop a strategy that employs low‐cost templating materials while ensuring high yield.

#### 4.2.3 Artificial Stacking

The artificial stacking method is becoming an advanced technique that can replace the conventional manual transfer approach. This newly introduced approach, named “autonomous robotic searching,” is based on artificial intelligence (AI).^[^ ^186^ ^]^ Here, the manual transfer technique uses an automated optical microscope to perform all the steps. First, all the layered flakes present on the substrate are scanned, and their locations are recorded. Then, a particular flake is selected according to the recorded location, and the robotic arm picks up the flake. This technique is comparatively faster, detecting around 400 monolayers and stacking four cycles of the designated 2D crystals per hour. However, the special setup used in this technique makes it costlier than the Manual transfer technique, which is a big concern from a commercialization point of view. This is a growing approach and needs to be explored more, which can bring revolution in the field of layered material science if such artificial stacking of (≈10000) layers becomes possible. Until now, this technique is at the laboratory level, and with more advancement, it can be successfully employed for integrating multilayered 2D HRs for practical applications. However, using artificial stacking, numerous 2D HR can be modeled by stacking 2D layers on a wafer as anode materials for microbatteries. On the same wafer, adding the metal (Li/Na/K) as a reference and filling of electrolyte in between them results in microbatteries that can help in analyzing electrochemical properties.^[^ ^187^ ^]^ In addition, the AI‐controlled method can also be employed to precisely design 2D HR materials directly on the Cu grid, which can be implemented for TEM to understand electrochemical properties and physical changes in 2D HR during their operation.^[^ ^188^ ^]^ Therefore, artificial stacking can emerge as an approach to fabricate these nano/micro‐batteries at a larger scale.

Overall, for the industrial applications of 2D HRs in batteries, the selection of the appropriate synthesis method is crucial. Like two sides of a coin, each strategy has its merits and demerits. Numerous factors, such as the ease in controllability of structure, cost‐effectiveness, quality of final material, and large‐scale productivity, highly influence the utility of any method in the industry. The radar plot in Figure 5b and **Table** **3** shows the complete comparison of these factors for all synthesis methods. They signify that the ball milling method is highly recommended for designing a few‐layered large‐scale 2D HR materials, whereas the solution‐based method is suitable for optimizing layer‐by‐layer ordered assemblies.^[^ ^131^, ^189^ ^]^ Other methods are limited to the laboratory scale. Furthermore, the commercialization of any battery device considers several steps when designing any electrode material, such as selecting the appropriate synthesis method, characterization, and optimization for desired quality, its mass production, the cost‐effectiveness of material and methods, and applicability to the device application (Figure 5c). In addition, the selection of the synthesis method is done according to the requirements of the structural and chemical properties of materials. For example, if BlackP needs to be implemented in electrode material, water‐based methods will not be applicable. Similarly, solution‐based methods and many others fail if the lateral assembly of HRs is required. In this context, Figure 5d displays the suitability of synthesis methods for synthesizing lateral and vertical 2D HRs. Notably, the hydrothermal and CVD methods have been approved for their suitability in designing both types of 2D HRs. Overall, the synthesis method plays a major role in determining the cost and efficiency of the final device.

**Table 3:** Merits and demerits of different synthesis techniques and their comparison toward applicability to synthesize 2D HRs for batteries.

Techniques	Merits	Demerits	Applicability for HRs
Manual transfer	Fewer defects	Not scalable, uncontrolled number of layers, time consuming	Not appropriate.
CVD	Controlled lateral and vertical HR, less defects	Lattice matching is required, inert atmosphere is needed, costly	Not preferable
ALD	Controlled synthesis, no defects	High cost, less efficient, low mass production	Not appropriate
Solution mixing	Scalable, cheaper, high yield, no special instrument required	Poor directional control, lateral HRs not possible	Highly preferable
Hydro/Solvothermal	Scalable, cheaper, less complex, Lateral and vertical assemblies are possible	Induced force required, high pressure needed, uncontrolled	Highly preferable
Ball milling	Scalable, cheaper	Only few layered composites are formed.	Preferable
Electrostatic self‐assembly	Scalable, ordered assembled structures	Low yield, unclean surfaces	Preferable
Template method	Porosity can be controlled by varying templates and easily applicable by using solution‐based methods	High cost due to special templates	Highly preferable for low‐cost templates

## 5 Experimental Validations of 2D HRs Efficiency in Monovalent Batteries

The 2D HRs are anticipated to serve as highly promising electrodes for rechargeable batteries, owing to the distinctive fundamental features outlined above. The primary advantages of these overwhelming characteristics include increased ionic diffusion kinetics, ion storage capacity, electrochemical reactivity, and cyclability. However, the performance of different M^+^ ion batteries may vary in response to these advantageous features, as they possess unique attributes and challenges. Consequently, to address specific challenges in batteries, it is crucial to select the appropriate 2D materials for the construction of efficient 2D HRs. They can be engineered in diverse sizes with tunable physicochemical properties, depending on the molecular structure of each layer, their interfacial features, and synthesis routes. Numerous experimental outcomes have corroborated the benefits of employing 2D HRs over individual layered structures for monovalent batteries by comparing their electrochemical performances. For such investigations, several physical quantities, such as specific capacity, current density, energy density, coulombic efficiency, and power density, are incorporated to assess the efficiency of batteries in terms of their rate performance, stability, and scalability. For reference, all quantities, accompanied by their concise definitions and corresponding formulas, are systematically tabulated in **Table** **4**.^[^ ^43^, ^69^, ^190^, ^191^, ^192^, ^193^, ^194^ ^]^ These parameters are derived by performing different electrochemical characterization techniques such as cyclic voltammetry (CV), galvanostatic charge–discharge (GCD), electrochemical impedance spectroscopy (EIS), and galvanostatic intermittent titration technique (GITT). A comprehensive summary of these techniques is also summarized in **Table** **5**.^[^ ^33^, ^70^, ^148^, ^192^, ^195^, ^196^, ^197^, ^198^, ^199^, ^200^, ^201^, ^202^, ^203^, ^204^, ^205^, ^206^ ^]^

**Table 4:** The table presents the different physical quantities, along with their definitions and formulas that are used to evaluate the electrochemical performance of batteries.

Physical quantity	Definition	Formulae [unit]		Refs.
Specific capacity (*Q*)	Electric charge or energy stored by electrode per unit mass	$Q=\frac{nF}{M}$ (mAh g^−1^)	*n* is the number of electrons participating in the reaction, *F* is Faraday's constant, and *M* is the mole weight of electrode active material.	[69, 190]
Coulombic efficiency	Ratio of the total charge extracted from the battery to the total charge supplied to it per charge‐discharge cycle	$\frac{Q_{d}}{Q_{c}}$	*Q*_d_ and *Q*_c_ are the discharge and charge capacities, respectively.	[191]
Diffusion coefficient	Mobility of M^+^ ions in the electrode or electrolyte medium	$D=\frac{4}{\pi \tau }\;{\left(\frac{m\;V_{B}}{MA}\right)}^{2}{\left(\frac{\Delta E_{S}}{\Delta E_{\tau }}\right)}^{2}$ (cm^2^ s^−1^)	τ is the duration of the current pulse, ∆*E*_s_ is the voltage change during a steady state, ∆*E*_τ_ is the voltage change when current pulse is applied, *A* is electrode surface area, *m* is mass loading, *M* is molar mass of active material	[192]
Open circuit voltage	The electric potential difference between the positive and negative terminals of the battery when no current is drawn	$OCV=-\;\frac{\Delta G}{ne}$ (V)	∆*G* is Gibb's free energy, and *n* is the number of electrons transferred.	[43]
Gravimetric (*E_G_*) / volumetric (*E_V_*) energy density	Amount of energy a battery can store relative to its weight or volume	$\;E_{G}=\frac{Q\;V}{Area}$ (Wh kg^−1^) $\;E_{V}=\frac{Q\;V}{Volume}$ (Wh cm^−3^)	*V* is the average voltage of the charge–discharge cycle.	[193]
Power density	How rapidly a battery can deliver the energy per unit weight or volume	$P=\frac{E_{G}}{\Delta t}$ (W kg^−1^) $P=\frac{E_{V}}{\Delta t}$ (W L^−1^) or (W cm^−3^)	∆*t* is the time taken by a battery to discharge.	[194]

**Table 5:** The table provides a summary of several experimental investigations useful in battery applications.

Technique	Analysis method	Parameters	Observations	Contribution toward battery performance	Refs.
Experimental investigations					
HRTEM	HRTEM/TEM imaging	Beam energy, vacuum, aperture sizes, diffraction/imaging mode, aberration corrections	TEM gives a pictorial micro/nanoscale view of materials. HRTEM provides atomic‐resolution images and interplanar distance	Detects morphology, SEI layer, defects, or any impurities in electrodes. Locates atoms and diffused ions, and confirms the incorporation of 2D layers in HR.	[195]
	STEM			Atomic positions of different layers and locate diffused M^+^ ions	[196]
	EELS		EELS spectra, EELS atomic resolution plots	Spectra evaluates characteristic energies of different layers of HR at the atomic level and changes in the electronic structure. The characteristic spectra detect ion migration.	[197, 198]
	SAED		Diffraction pattern and corresponding inverse fast Fourier transform	Identify crystal planes, lattice structure, interplanar distance, crystallinity	[195]
XRD	–	Wavelength, detector properties	Grain size, lattice parameters, phase, type of crystal structure	Determine changes in interplanar spacing, defects, and phases during reaction mechanisms.	[148, 199]
XAS	EXAFS/XANES	Energy of incident X‐ray photons, detection mode, beamline, and its spot size alignment	Coordination environment, oxidation, and electronic structure, dynamic behavior	Determine phase and oxidation state change during the reaction, track degradation mechanisms of electrodes, and detect the composition of the SEI layer. Help in optimizing electrolytes and electrodes.	[200]
Raman	–	Wavelength, grids, optical arrangements	Molecular vibrations, crystallinity, defects, deformations, phase structures	Optimize electrode material and the changes (phase, crystallinity, deformations, etc.) during the cell performance.	[196]
XPS	–	Vacuum, energy range, number of scans, source of X‐rays	Detects elemental composition, chemical state	The chemical composition of electrode materials can be optimized; height profiling can be done to study SEI layer components, and in/ex situ studies provide detailed composition changes during cycling.	[196]
AFM	Height profiling	Resonant frequency, tip–sample distance	Orientation and thickness of different layers	Detects volume and morphology changes of electrodes.	[70, 201]
	Scanning tunneling microscopy	Voltage between probe and sample	Defects, electron density distribution, atomic level resolution	Observes migration of M^+^ ions	[202, 203]
	Kelvin probe force microscopy	Applied bias, tip–sample distance	Work function and surface potential mapping	Charge distribution, local electronic properties, and electrochemical potential changes during battery operation.	[204]
	Force spectroscopy/ nanoindentation	Indentation depth and force, tip geometry	Mechanical strength and strain	Determines stress induced during charging–discharging, flexibility (mechanical strength), volumetric changes in electrodes and separators	[205]
Electrochemical measurements	CV	Voltage window, sweep rate	Current vs. voltage	The redox activity of the electrode, reversibility, diffusion kinetics by peak shifting, and the area enclosed by the curve give specific capacity.	[33]
	GCD	Voltage window, specific current rate	Voltage vs time, voltage vs capacity	Battery capacity, cycle stability, coulombic efficiency, rate capability, voltage hysteresis (overpotentials and polarizations)	[199]
	EIS	Frequency and amplitude of the wave	Nyquist plot, fitted circuits representing different impedances.	Resistance offered by different components toward mobile M^+^, ionic conductivity	[206]
	GITT	Current pulses, relaxation time, mass loading, molar mass	Voltage response as a function of time	Change in diffusion coefficient for M^+^ in HR, overpotentials	[192]

Note: HRTEM = high‐resolution transmission electron microscopy, STEM = scanning tunneling electron microscopy, EELS = electron energy loss spectroscopy, SAED = selected area electron diffraction, XRD = X‐ray diffraction, XAFS = extended X‐ray absorption fine structure, XANES = X‐ray absorption near edge structure, XPS = X‐ray photoelectron spectroscopy, AFM = atomic force microscopy, GCD = galvanostatic charge–discharge, CV = cyclic voltammetry, EIS = electrochemical impedance spectroscopy, GITT = galvanostatic intermittent titration technique.

Rechargeable LIBs are well known for their performance at commercial as well as laboratory scales. Layered graphite with an interlayer spacing of 0.334 nm has been recognized as a prominent anode material for LIBs, tailored by its excellent cycle stability, low operating potential, and high electronic conductivity. Still, its performance is limited by its poor specific capacity (372 mAh g^−1^) due to fewer active sites. Furthermore, Gr, with a dual active surface, owns a 740 mAh g^−1^ theoretical specific capacity for LIBs but is practically restricted by an issue of restacking. Likewise, MXenes containing redox‐active functionalities (─O, ─OH, and ─F) and high conductivity (6.76 × 10^5^ S m^−1^) can provide capacities much better than carbons but are again restricted due to restacking issues.^[^ ^155^ ^]^ Subsequently, hybridizing Gr/GO/rGO or MXenes with the other 2D materials, TMDs, and TMOs forms 2D HRs with weak vdW interaction and resolves the re‐stacking problem. Also, the redox‐active transition metal‐based compounds, such as TMDs or TMOs enhance electrochemical activity and significantly improve the overall specific capacity of Gr or MXene layers. Moreover, the poor electronic conductivity of TMDs or TMOs is compensated by adding Gr or MXenes. Such symbiotic structures turn out to be the perfect hosts for M^+^ ions, providing better structural stability and reversibility to the electrodes. Gogotsi et al. emphasized the effect of hybridizing different concentrations of TMO and MXene (H‐0.5 sample contains a higher concentration of MXene and H‐4 with a lower concentration) on Li‐ion storage.^[^ ^207^ ^]^ Higher Ti_3_C_2_T_x_ content was observed to give superior mechanical flexibility and conductivity to the material, whereas a higher amount of Co_3_O_4_ improves the specific capacity of the resultant anode material. The specific capacity of H‐4.0 with a higher Co_3_O_4_/MXene ratio is relatively larger than that of a lower ratio (H‐0.5). The remarkable specific capacity of H‐4 is due to the larger content of Co_3_O_4_, which has a high theoretical specific capacity (890 mAh g^−1^) compared to MXene (≈500 mAh g^−1^).^[^ ^207^ ^]^ Hence, TMOs can improve the specific capacities of MXenes when combined in a specific ratio. Therefore, optimizing the ratio of each layered material participating in HR formation is necessary. Otherwise, a higher amount of any of them can degrade the performance due to their unfavorable properties. To present the concept clearly, an example of V_2_O_5_/MXene HR can be taken.^[^ ^155^ ^]^ HRs with different wt% of V_2_O_5_ having high theoretical capacity were synthesized, and it was found that the gravimetric capacity for hybrids having 40 wt% of V_2_O_5_ was maximum and decreased when the concentration increased to 60 wt%. The reason is that the increased content of V_2_O_5_ in 60 wt% leads to large volumetric changes and, hence, low resultant capacity. Apart from this, V_2_O_5_/MXene HR with different directional assemblies (vertical and horizontal) were synthesized over the current collector by mechanically pressing the hydrogel. Among these, the vertical assembly provides extra channels for electron and ion diffusion, along with a robust and binder‐free structure, providing the advantage of high mass loading. Depending on the directional arrangement of the vertical assembly, the benefits of high gravimetric capacity, low resistance, and high diffusion coefficient (*D*) can be achieved.^[^ ^155^ ^]^

GDY is a newly emerging thin structured (≈1–2 nm) carbon nanomaterial for 2D HRs, possessing 18‐C cavities (0.54 nm), highly active triple bonds, attached functional groups (C═O, C─OH, and ─COOH), π‐conjugated surface, triangular pores (≈0.25 nm), high work function (5.53 eV), good electrical conductivity (2.5 × 10^−4^ S m^−1^), and exceptional chemical stability, thereby providing abundant active sites for Li^+^ ions.^[^ ^195^, ^208^ ^]^ Recently, TiC_2_T_x_/GDYO HR has been synthesized for LIBs, where incorporation of GDYO led to an increase in the interplanar spacing of densely packed TiC_2_T_x_ from 1.33 to 1.5 nm.^[^ ^195^ ^]^ The integrated HR attained a specific capacity of 1204.6 mAh g^−1^, higher than that of the Ti_3_C_2_T_x_ (336.0 mAh g^−1^), GDYO (306.8 mAh g^−1^), physical mixture of Ti_3_C_2_T_x_ and GDYO (755.4 mAh g^−1^). This reflects that the intercalation of GDYO layers is beneficial in enhancing the performance of Ti_3_C_2_T_x_ by enlarging interlayer spacing, in‐built electric field, and providing more Li^+^ ion storage sites.^[^ ^195^ ^]^ Beyond increased specific capacities, the strategically designed robust assemblies of HR active materials are helpful in providing longer life to batteries by reducing pulverization. In this context, the lateral/vertical HR of GDYO/BlackP was synthesized by the ball‐mill process to mitigate the pulverization in BlackP. The lateral contact of GDYO with BlackP is realized by sp^2^ hybridizing C from GDYO and P from BlackP, while the vertical junctions were obtained by P─O─C covalent bonds. Strong covalent coupling and intrinsic properties of GDYO result in alleviating the limitations of BlackP and ensure accelerated reaction kinetics and structural stability during reversible reactions. Meanwhile, the engineered structure improved the electrical conductivity of BlackP (80 S cm^−1^) to 100 S cm^−1^. After 1000 cycles, the GDYO/BlackP HR maintained a capacity of 602.6 mAh g^−1^, much higher compared to BlackP (50.4 mAh g^−1^).^[^ ^208^ ^]^

Despite similar physical and chemical properties of Na^+^ to Li^+^ ions, such as low half‐reaction redox potential and monovalent charge, the SIBs encounter pronounced constraints compared to LIBs. The larger ionic radius of Na^+^ (1.02 Å) than Li^+^ (0.76 Å) ions and higher molecular weight are the primary issues that limit the kinetics of Na^+^ ions, followed by phase instability and intermediates formation.^[^ ^209^, ^210^ ^]^ Interestingly, while graphite serves as the commercialized anode material for LIBs, delivering a capacity of 372 mAh g^−1^, it behaves entirely differently when assembled for SIBs, offering a markedly lower theoretical specific capacity of just 35 mAh g^−1^.^[^ ^211^, ^212^ ^]^ The disparity arises from the high positive enthalpy of Na^+^ ions associated with the formation of NaC_6_ and NaC_8_ (+20.8 and +19.9 kJ mol^−1^) in graphite, unlike Li^+^ and K^+^ ions, which form LiC_6_ and KC_8_ intermediates with negative enthalpies (−16.4 and −27.5 kJ mol^−1^).^[^ ^213^, ^214^ ^]^ Consequently, the intercalation of Na^+^ ions is unfavorable in graphite with an interlayer spacing of 0.334 nm and a high *E_SD_* barrier (0.12 eV).^[^ ^215^, ^216^ ^]^ Thus, to facilitate the intercalation of Na^+^ ions, the graphite layers can be expanded, or their interfacial properties can be tuned by incorporating other materials to achieve a more robust structured hybrid material. For satisfactory results, HRs with extensive overlapping areas are highly preferred. To illustrate this, Wang et al. have designed SnS_2_/Gr HR by covalently bridging the SnS_2_ ultrathin layers on Gr sheets.^[^ ^39^ ^]^ The controlled isotropic growth of SnS_2_ amorphous seeds over carbon matrix along (001) planes results in their face‐to‐face bridging (C─S─Sn bonds) to attain a robust structure. This HR with an interlayer spacing of 5.9 Å allows easy penetration of Na^+^ ions and helps to achieve 259 mAh g^−1^ capacity at a current density of 20 A g^−1^. The performance is much higher than that of SnS_2_, which exhibited nearly zero capacity at this current density. Similarly, Zhang et al. have synthesized MoSe_2_/Gr composite by the hydrothermal route for Na^+^ ion storage. As synthesized hybrid with a hierarchical structure was utilized as an anode and shows remarkable cycling stability (up to 200 cycles at 0.5A g^−1^ and 1A g^−1^) along with high‐rate performance (380 mAh g^−1^ at 1 A g^−1^) in comparison to bare MoSe_2_ (469 mAh g^−1^ at 100 mA g^−1^ and stable up to 70 cycles). The performance improvement is attributed to the decline in diffusion length, increased conductivity, and larger contact area between the electrode and electrolyte as a result of the synergistic effect. In a similar fashion, several other HRs combining TMDs, MXenes, and TMOs with Gr have been realized for SIBs.^[^ ^51^, ^156^ ^]^

**Figure 6:**
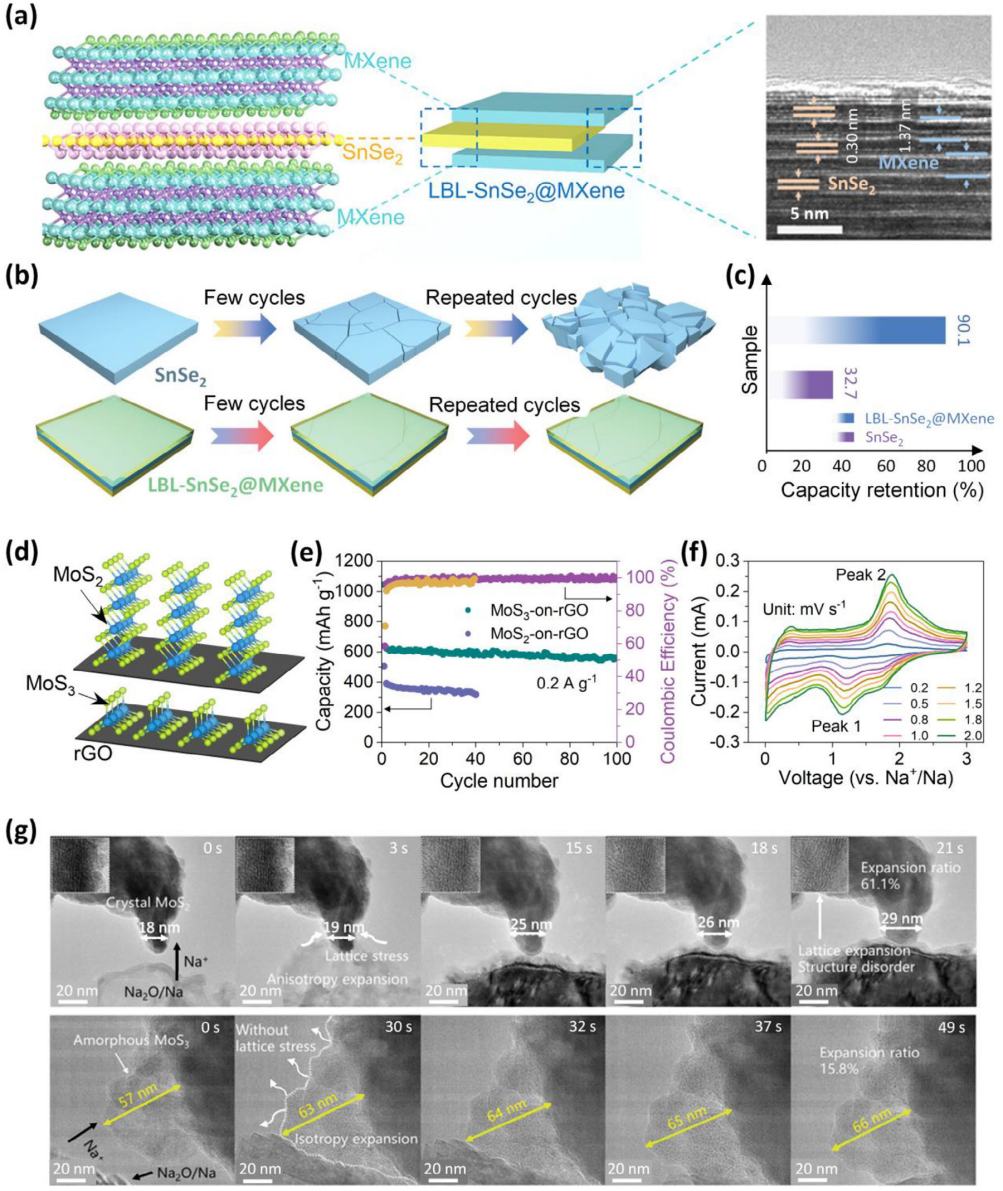
a) The 3D illustration of layer‐by‐layer assembly of SnSe_2_/MXene HR with the magnified view representing their crystal structures (left side) and HRTEM image (right side). b) The schematic illustrates that hybridizing SnSe_2_ with MXene prevents it from structure degradation. c) The bar graph shows improved capacity retention due to a stable SnSe_2_/MXene HR. Reproduced with permission.^[^ ^217^ ^]^ Copyright 2023, Wiley. d) Schematic diagram of MoS_2_‐on‐rGO and MoS_3_‐on‐rGO HRs. e) The plot shows better capacity and stability of MoS_3_‐on‐rGO over MoS_2_‐on‐rGO HR, attributed to amorphous features of MoS_3_. f) CV plots at different sweep rates for MoS_3_‐on‐rGO. g) In situ TEM images after different charging times demonstrate less volume expansion in MoS_3_‐on‐rGO (15.8%) than in MoS_2_‐on‐rGO (61.1%). Reproduced with permission.^[^ ^115^ ^]^ Copyright 2023, Wiley.

In addition, the approach of HR construction is highly favored in alloying anodic materials, with a primary focus on mitigating the pulverization effect and volumetric changes. The mechanically stable 2D materials, such as Gr and MXene, are selected to integrate HR with alloying‐based materials. From this perspective, Kong et al. have grown layer‐by‐layer assembly of SnSe_2_ and widely spaced MXene (LBL‐SnSe_2_@MXene) utilizing terminal group‐oriented self‐assembly, as shown in **Figure** **6**a.^[^ ^217^ ^]^ Notably, the alloying properties of SnSe_2_ induce drastic volumetric changes during cycling, which can lead to electrode delamination after repeated cycles (Figure 6b). However, with the support of mechanically stable and flexible MXene layers, the capacity retention of SnSe_2_ is improved from 32.7% to 90.1% after 150 cycles, as depicted by the bar plot in Figure 6c. Similarly, Guo and his coworkers prevented the pulverization of BlackP by hybridizing it with flexible MXene, which helped accommodate high volumetric changes caused by the diffusion of Na^+^ ions.^[^ ^77^ ^]^ They also investigated the advantage of HR over the physically mixed composite of 2D layers. After 100 cycles, the capacity of the mechanically mixed BlackP/MXene sample dropped to 171 mAh g^−1^ compared to 467 mAh g^−1^ of P/MXene HR, which is ascribed to the high‐volume expansion in BlackP (250%) compared to that in HR (120%).

Noticeably, hybridizing more than two distinct layers can be further fruitful in improving the performance with the add‐on properties of each layer. To configure this, Tang et al. have grown WS_2_/MoS_2_ HR between the MXene lamellae layers and attained robust WS_2_/MoS_2_/MXene HR. The trilayered WS_2_/MoS_2_/MXene HR performs with a capacity of 505.2 mAh g^−1^, much higher than that of bilayered MoS_2_/MXene (311.6 mAh g^−1^) and WS_2_/MXene (261.9 mAh g^−1^) HRs.^[^ ^145^ ^]^ The robust 3D conductive network is responsible for a large number of active sites and facile interfacial kinetics, which further contribute to decreased ohmic polarization for Na^+^ ions. The other researchers have also observed similar progress in HRs with more than two distinct layered structures.^[^ ^74^, ^218^ ^]^

MoS_2_, when used alone as an anode material versus sodium as a counter, cannot retain the capacity for a longer time as a result of high volumetric changes due to intercalation‐conversion reactions. When MoS_2_ is incorporated between MXene layers, it can lead to a stable anode material exhibiting an astonishing reversible capacity of 315 mAh g^−1^ at 0.2 A g^−1^ and 220.0 mAh g^−1^ after 1000 cycles at 2.0 A g^−1^ when compared with MoS_2_.^[^ ^93^ ^]^ In addition, modifying the crystal structure of MoS_2_ by introducing defects, functionalities, or amorphousness can help in further reducing volume changes and improving stability. In this regard, MoS_3_ with amorphous properties is grown over the rGO layer by an isotropic growth process to compare its performance with that of crystalline MoS_2_.^[^ ^115^ ^]^ At different temperatures, the growth of decomposed precursor over rGO sheets leads to crystal and amorphous seeds of MoS_2_ and MoS_3_. The amorphous MoS_3_ nuclei favor isotropic growth and tend to form a 2D cover‐on‐rGO HR, whereas that of crystalline MoS_2_ follows anisotropic growth that gives nanosheets standing perpendicularly, as displayed in Figure 6d. For Na^+^ ion storage, MoS_3_‐on‐rGO performs with higher cyclic stability and better reversibility than MoS_2_‐on‐rGO (Figure 6e). The CV curves for MoS_3_‐on‐rGO at different rates show consistent peaks (Figure 6f) with high pseudocapacitive contributions (≈76%–92%) that result in superior electrochemical kinetics. During the sodiation process, the disordered nanostructure of MoS_3_‐on‐rGO possesses internal free volume that relieves the strain and shows a low volume expansion (only 15.8%) compared to 61.1% of MoS_2_‐on‐rGO HR, proven by in situ TEM images (Figure 6g).

For the past few years, P, mainly BlackP, due to outstanding electronic conductivity (≈10^3^ S m^−1^) and high theoretical capacities, has emerged as a significant candidate for anode material in alkali M^+^ ion storage. However, its structural and chemical instability is the limitation restricting its use in batteries. Capping it with other layered materials, such as Gr, hBN, and MXene, can significantly improve the stability. The 2D layered structures have been widely explored by incorporating BlackP in them and achieved enhanced results. BlackP/Ti_3_C_2_ HR shows remarkable performance and stability with 1112 mAh g^−1^ at 0.1 A g^−1^ after 500 cycles.^[^ ^70^ ^]^ The surface functionalities attached to MXenes also tune chemical reactions that further enhance the stability of BlackP. The fluorine terminations at the surfaces of MXene facilitate the formation of a fluorine‐rich SEI layer. The stable SEI layer improves the reversibility of ions, i.e., stabilizing the coulombic efficiencies. The resultant MXene/BlackP HR exhibits almost 50% less volume changes (obtained due to pulverization) than the pristine BlackP and delivers an appreciable capacity of 340 mAh g^−1^ at 1 A g^−1^ with a capacity retention of 87% after 1000 cycles.^[^ ^77^ ^]^ In addition, most of the transition metal‐based materials follow conversion reaction processes. The resultant performance often leads to large voltage hysteresis, resulting in fast capacity and energy loss during reactions. It is well established that HR formation can improve the conductivity of 2D materials and, hence, help mitigate voltage hysteresis issues. In this regard, Cai et al. compared the charge–discharge and dQ/dV curves for Cu_2_S and Cu_2_S/Cu_5_FeS_4_ HR to study the change in voltage hysteresis.^[^ ^199^ ^]^ The voltage hysteresis was observed in Cu_2_S as well as its HR (Cu_2_S/Cu_5_FeS_4_) due to sluggish kinetic diffusion of Na^+^ ions during multiphase complex conversion reactions. However, at 5 A g^−1^, its value for HR was only 0.65 V, much lower than that of Cu_2_S (1.06 V), mainly attributed to the increased conductivity of HR. Such newly emerging HR materials need to be explored more for Na^+^ ion storage.

K^+^ is another monovalent ion that has also been appreciated as a suitable ion for batteries due to its abundance, almost no toxicity, less redox potential, and higher ionic diffusion in electrolytes than Li^+^ ions. These properties are attributed to their low interaction with solvents and anions. However, the large ionic radius (1.38 Å) limits its kinetic properties and is the reason for its moderate theoretical capacity (279 mAh g^−1^) with graphite. In comparison to Na^+^, K^+^ ions face less *E_SD_* barrier for anodes. Due to its large ionic radius, anode materials with higher interlayer spacing are the primary requirement for K^+^‐based ESDs. Thus, TMDs with inherited large interlayer spacing can be promising anode materials and are widely investigated. But sluggish kinetics of K^+^ ions in TMDs, ascribed to their poor conductivity, restrict their use. However, using them in HRs can be a conducive solution for satisfactory performance. In a recent research, Ma et al. combined MoSe_2_ and MoS_2_ with N‐doped layered carbon (NC).^[^ ^91^ ^]^ They successfully observed that the enhanced rate performance of both HRs (130 mAh g^−1^ at 10 A g^−1^ for MoS_2_ on NC and 247 mAh g^−1^ at 1 A g^−1^ for MoSe_2_ on NC) is due to increased K^+^ diffusion as a result of synergistic effects. Following a similar strategy, Cui et al. have grafted bilayer MoS_2_ over N, S‐doped carbon, and Ti et al. have separately synthesized MoS_2_/Gr composite for the K‐ion capacitor.^[^ ^219^, ^220^ ^]^ The capacitor exhibited a specific capacity of 451.2 mAh g^−1^ at 0.1 A g^−1^ and found stability up to 20000 cycles with only 0.0013% fading per cycle. Benefitting from the inherent properties of each layer in SnS/MoSe_2_/Gr HR, Wang et al. obtained an anode material that reflects high conductivity, excellent redox activity, and good reversibility, thereby buffering the volume changes during charging–discharging. The SnS/MoSe_2_/Gr assembly exhibits the highest potassium storage capacity (480 mAh g^−1^) compared to MoSe_2_ (306 mAh g^−1^), SnS (295 mAh g^−1^), MoSe_2_/Gr(390 mAh g^−1^), and SnS/Gr (300.7 mAh g^−1^).^[^ ^196^ ^]^ Similarly, researchers have put their efforts into preparing many HR combinations for achieving advantageous collective properties, giving significant results.^[^ ^221^, ^222^ ^]^

**Figure 7:**
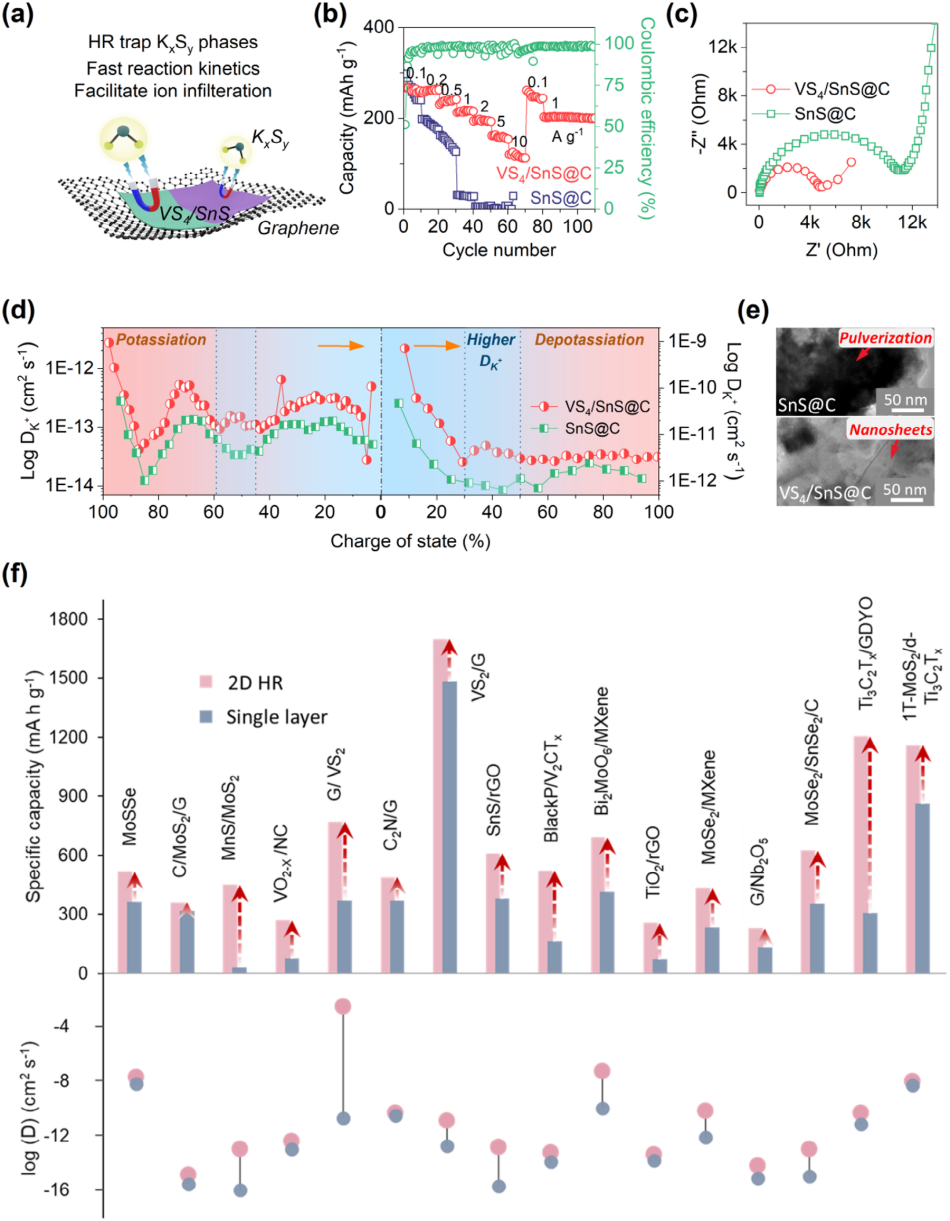
a) The schematic illustration showcasing the polar 2D VS_4_/SnS HR traps K*_x_* S*_y_* phases and prevents them from dissolving in the electrolyte. Half‐cell electrochemical performance of 2D VS_4_/SnS@C HR anode for PIB demonstrates: b) improved rate capability, c) decreased resistance, and d) enhanced *D* values than that of SnS@C. e) Ex situ TEM after 100 cycles represents the pulverization in SnS@C, which diminished in VS_4_/SnS@C HR. Reproduced with permission: Copyright 2021, Wiley.^[^ ^223^ ^]^ f) The comparative graph shows the calculated *D* values (lower panel, dumbbell chart) and specific capacities (upper panel, bar graph) for individual 2D structured materials (blue color) and their corresponding 2D HRs (pink color). Data collected from refs. [82, 94, 102, 195, 224, 225, 226, 227, 228, 229, 230, 231, 232, 233, 234, 235].

Notably, the construction of HRs also alters several chemical properties of each layer toward the reaction mechanism, which can be beneficial in stabilizing the electrode. Alloying‐conversion type electrodes typically follow irreversible reactions, leaving unwanted nonreactive components. Such components get dissolved into electrolytes, and eventually, there is active mass loss from electrodes. Cao and his co‐workers have used a strategy to prevent such loss of active material caused by dissolving intermediate K*_x_* S*_y_* in the electrolyte.^[^ ^223^ ^]^ They designed VS_4_/SnS@C HR by grafting the homogenous layer composed of polar VS_4_ and SnS over the Gr layers, as shown in **Figure** **7**a. Here, VS_4_ inherits a chain‐type structure of parallel quasi‐1D V^4+^(S_2_^2−^)_2_ with double unsaturated bridging (S_2_)^2−^ moieties around the V atom linked by vdW bonds. The diffused intermediate K*_x_* S*_y_* can be trapped and localized between the V_4_^+^(S_2_^2−^)_2_ chains through intense vdW interactions of unsaturated (S_2_)^2−^ moieties, preventing the loss of active materials during repeated cycling. After 3000 cycles, the capacity decreased only to 227 mA h g^−1^ from 243.5 mA h g^−1^ at 0.5 A g^−1^ (98.3% capacity retention).^[^ ^223^ ^]^ Even at higher rates, it notably shows a superior performance with 122.7 mAh g^−1^ capacity at 10 A g^−1^, and also at 0.1 A g^−1^, an excellent reversible performance was recovered after testing the same cell at ultrahigh currents (Figure 7b). Also, the tremendous electrochemical activity of VS_4_/SnS@C HR is reflected by the smaller value of charge transfer resistance (*R*_ct_: 4530 Ω) than that of SnS@C (*R*_ct_: 13500 Ω) composite (Figure 7c). Furthermore, the enhanced diffusion kinetics of K^+^ ions in HRs was observed by the GITT technique, where calculated *D_K+_* values (Figure 7d) were larger for VS_4_/SnS@C than SnS@C. The rising trend in blue boxes reflects the entrapment of the K*_x_* S*_y_* components during the conversion reaction. The TEM images (Figure 7e) show pulverization in SnS@C after 100 charge‐–discharge cycles, which is clearly not visible for transparent layered VS_4_/SnS@C HR, validating the reversible conversion reactions of Sn^0^/K*_x_* S*_y_*.^[^ ^223^ ^]^

As highlighted above in the theoretical section, the diffusion barriers for moving M^+^ ions are minimized in HR, attributed to BIEF and other chemical changes. Such improved diffusion of ions can also be investigated by electrochemical analysis. Experimentally, both GITT and EIS electrochemical techniques have been widely performed to estimate the *D*_M+_ values of M^+^ ions in electrodes during the charging–discharging process. Drawing from multiple experimental investigations, the comparative plots (Figure 7f) illustrate the calculated *D*_M+_ values (dumbbell plot at the lower side) for monolayers (violet) and their respective HRs (pink).^[^ ^82^, ^94^, ^102^, ^195^, ^224^, ^225^, ^226^, ^227^, ^228^, ^229^, ^230^, ^231^, ^232^, ^233^, ^234^, ^235^ ^]^ The monolayers exhibit lower values than those of HRs, aligning with the above‐mentioned discussion. Furthermore, the resultant elevated capacities for the same monolayers (violet) and their respective HRs (pink) are shown by the bar plot in the upper section.^[^ ^82^, ^94^, ^102^, ^195^, ^224^, ^225^, ^226^, ^227^, ^228^, ^229^, ^230^, ^231^, ^232^, ^233^, ^234^, ^235^ ^]^ This demonstrates that the performance of HR materials is higher than that of their monolayer counterparts, attributed to their synergistic effects and their intrinsically modified fundamental properties. The improved diffusion kinetics increases the utilization of electrode active material, resulting in enhanced specific capacities of HRs. Therefore, the strategy of imposing 2D HRs as anode materials proposes a functional way of improving the battery performance of all monovalent ions. It is noteworthy that most studies focus on electrochemical investigations in half‐cell assemblies, which are not utilized in real‐world applications. For the market, it is crucial to incorporate full cells with a balanced N/P ratio, adhering to standard protocols of weight, cost, efficiency, and size. To assess the true potential of HR materials in batteries, the electrochemical analysis of full‐cell pouch cells providing gravimetric/volumetric performance should be conducted. For large‐scale applications, such as electric‐vehicles, the full‐cell battery device needs to reach 80% state of charge within 15 min, as suggested by the U.S. Advanced Battery Consortium (USABC). Comprehensive research on HRs can contribute toward meeting this challenge, enabling their use in a wide‐range of large scale applications.

## 6 Current Challenges for 2D HRs in Battery Applications

From the comprehensive assessment in the previous sections, it is clear that the scientific community has recognized the potent capabilities of 2D HRs through both theoretical and experimental approaches. Such investigations have provided a deep understanding of the fundamental phenomena driving the strong performance of HR anodes in monovalent batteries. The modified electrical properties in HR materials facilitate ion diffusion and stabilize the structure, thereby enhancing battery rate capability and stability. The increased capacity indicates that a full‐cell device can be designed with comparatively less anode material while maintaining the N/P ratio. This improves the gravimetric and volumetric energy densities, highlighting the practicality of 2D HRs as anodes in batteries. Despite their advantages, these materials still show limited adoption in the industry due to several challenges they face. This section reveals some of the significant challenges encountered by 2D HRs and outlines the approaches that can help meet the desired requirements.

### 6.1 Scalable Synthesis Pathways for Battery Production

Large‐scale production of HR materials for batteries presents a significant challenge, particularly in achieving an ordered layer‐by‐layer assembly. As discussed above, each synthesis method has its own advantages and disadvantages from a manufacturing perspective. A method that excels in quality may fall short in scalability and vice versa, as summarized in Table 2. The mechanical transfer and vapor deposition methods are highly suitable for achieving pure layer‐by‐layer HR materials; however, they are impractical for large‐scale battery production due to their prolonged and tedious processes. Alternative approaches such as solution‐based assembly, hydro/solvothermal method, and ball milling can be used effectively for the mass production of electrode material but cannot control multilayer assemblies with more than two different monolayers. In this context, electrostatic self‐assembly can be a viable choice but is also limited by the high cost of charge‐generating polymers. Therefore, finding a new scalable approach for synthesizing highly ordered HR anodic materials becomes imperative, considering cost as a crucial parameter.

To discover a cost‐effective path, a technique named “microfluidization associated with roll‐to‐roll padding” has recently been introduced to obtain the heterostructured flexible electrodes ofGr and MoS_2_.^[^ ^236^ ^]^ Microfluidization is a scalable and environment‐friendly method in which a high pressure (≈207 MPa) is applied to the solution of material and forces the liquid to pass through a microchannel of diameter ≈100 µm. Repeating this process multiple times exfoliates the material, forming a dispersion of 2D Gr or MoS_2_. By further rolling the textile from these dispersions sequentially, they obtained MoS_2_/Gr HR‐coated fiber for supercapacitors. A similar process can be incorporated for batteries. Another possible solution to reduce the cost can be the utilization of commercially available, cheaper 2D materials and then coating them directly on current collectors by spray coating or inkjet printing. This reduces several steps of electrode fabrication and hence will reduce the cost. Similarly, electrodeposition methods can be incorporated, which again grow the HR layers over current collectors, thereby avoiding the costly synthesis steps. To further reduce the cost of materials, cheaper precursors, including earth‐abundant oxides and chlorides, can be used instead of pure elemental forms For example, (e.g., to prepare MoS₂ by the CVD method, instead of the Mo element, their alternative precursors can be utilized).

In addition to cost‐effectiveness, environment‐friendly processes that do not incorporate toxic materials and consume minimal energy are highly preferable in industry. Fabrication of certain 2D materials requires special environments/conditions, increasing complexity in synthesizing HR. For example, BlackP, which has an astonishing theoretical capacity for all M^+^ ions, is found to be sensitive to environmental factors and requires an inert atmosphere. Therefore, these are restricted for HR synthesis when following aqueous solution‐based systems. Also, the synthesis process of many 2D materials, such as MXenes and Gr, involves highly concentrated acids such as hydrofluoric acid (HF), sulfuric acid (H_2_SO_4_), and nitric acid (HNO_3_). These hazardous chemicals require strict safety protocols, including the use of personal protective equipments (PPE) and controlled environmental conditions. These chemicals pose risks to human health and the environment due to their corrosive and toxic nature, thereby constraining their widespread research and practical applicability. Thus, the utilization of such 2D materials for HRs has become a very tedious task for researchers due to inappropriate and complex synthesis procedures. Although to resolve these issues, some environmentally friendly synthesis mechanisms have been proposed. For example, MXenes can be etched using electrochemical, alkali, and molten salt etching methods, which are HF‐free methods. Similar solutions can be explored to tackle the issues associated with the synthesis methods of other layered materials. A clear pathway for their incorporation needs to be presented in the research field without compromising the yield, quality, and stability of individual materials, which are essential factors from an industrial point of view. This makes the proposal of an appropriate novel synthesis technique an urgent need to realize appreciable properties of layered HR materials in laboratories as well as industries.

### 6.2 Unexplored Areas of Research

Hundreds of 2D layered materials have been discovered in the last two decades, and research continues to approach thousands of them. However, only a few have been utilized for HR assemblies. For instance, MXenes represent hundreds of different structures based on their functionalities and atomic features. Yet only a handful have been incorporated into HR synthesis, often limited by inappropriate inherent properties such as the type of functional group attached, band structure, and conductivity. It is also well known that most TMOs are naturally occurring stable compounds. Among these, many‐layered TMOs exist, but their use as 2D structured materials in batteries is quite limited. This limitation is due to their anisotropic growth, which complicates the control of their growth in a single dimension, making them more favorable as hierarchical HRs instead of 2D HRs. If leveraged as 2D layers, they could enhance electrochemical activity and lower costs compared to other materials like MoS_2_ and MXenes, which require a special inert environment at high temperatures and toxic precursors.^[^ ^237^ ^]^ Apart from this, numerous 2D materials, such as graphyne and transition metal carbochalcogenides, have been recently discovered, but these have not yet been used in HR assemblies for batteries. Furthermore, designing high‐entropy 2D materials that combine the properties of more than five different elements into a single 2D material has recently become a hot topic among researchers. The potential of high‐entropy 2D TMDs and MXenes has been explored for various applications. For example, Co_0.6_(VMnNiZn)_0.4_PS_3_ nanosheets have been studied for the hydrogen evolution reaction,^[^ ^238^ ^]^ (Ti_1/5_V_1/5_Zr_1/5_Nb_1/5_Ta_1/5_)_2_AlC derived 2D MXene in LIBs,^[^ ^239^ ^]^ and Ti_1.0_V_0.7_Cr_0.05_Nb_1.0_Ta_1.0_AlC_3_ based MXene in supercapacitors.^[^ ^240^ ^]^ Nevertheless, many such combinations of metals remain undiscovered and invite the research community for their exploration in battery applications.

A complete understanding of the factors restricting the utility of all 2D materials is open‐ended research. Systematic evaluation and comparison of these materials based on performance metrics are crucial for establishing a coherent research direction. Thus, scrupulous theoretical as well as experimental investigations about the interaction of ions with molecules of each layer and finding favorable conditions for the composition of stable HRs are needed, which can assist in selecting the appropriate layered materials. Incorporating in situ characterization techniques like in situ dilatometry, XPS, XRD, and TEM, altogether can give real‐time information on volume expansion, compositional changes, structural changes, and electrochemical changes occurring in HRs during ongoing reactions. For instance, Cao et al. employed in situ XRD to thoroughly understand the phase transition and reaction mechanism of VS_4_/SnS@C HR anode during potassiation/de‐potassiation.^[^ ^223^ ^]^ During potassiation, the crystal planes corresponding to the SnS and VS_4_ gradually vanished, and the planes corresponding to Sn^0^ and K_2_S appeared, demonstrating the alloying‐conversion reaction of HR materials. Moreover, during de‐potassiation, the appearance of planes corresponding to KSn and K_4_Sn_23_ further indicates the occurrence of alloying‐type reactions. Such findings can substantially help researchers optimize the performance of different HR materials in batteries.

### 6.3 Unprecedented Mechanisms Revealing Principles of Interactions

Theoretical calculations have revealed the grounds behind the boosted performance of HRs by estimating BIEF and energies associated with adsorption and diffusion processes. Most of these studies have focused on the behavior of HRs composed of two distinct vertically stacked monolayers. However, there is a noticeable gap in studies exploring the direction of BIEF, favorable adsorption and desorption sites, and other critical factors for HRs comprising more than two different layers.^[^ ^74^ ^]^ Along with this, little attention had been given to examining the change in performance for diverse configurations, such as lateral and vertical assemblies.

A broader and more refined perspective is needed to gain a clear understanding of the fundamental characteristics of such HR, which can further outline their electrochemical behaviors in batteries. This should include clear insights into how specific properties of each component contribute to the overall functionality of HR, the interactions between different layers, and their interplay with intercalating ions. For a few HR configurations, such as lateral (when edges of layers are connected) and vertical (when face‐to‐edge are bounded), more focused studies are needed to elucidate how these structures can be confined in specific directions, offering valuable guidance for their practical application in batteries.

### 6.4 Theoretical versus Experimental Differences

Hundreds of reports demonstrate the benefits of different 2D HR assemblies for batteries. After summarizing these reports, it can be confirmed that most of the combinations are only theoretically investigated and claim the existence of such HR. Nevertheless, many of them have not been experimentally achieved, specifically for battery applications. Therefore, it is crucial to identify possible conditions for achieving their stable structures such that they can be successfully utilized for device fabrication. In addition to this, countless experimental studies have reported the materials exhibiting performance and capacities surpassing even their nearby theoretical values. These findings call for theoretical validations to substantiate such claims and to establish a clear understanding of the fundamental principles driving enhanced performance.

On the other hand, there are reports in which materials are unable to meet theoretical expectations. It is well known that theoretical calculations are proposed by considering ideal conditions. However, the practical implementation of the same system cannot obey the same conditions everywhere. During the operation of a battery, numerous conditions, such as temperature, humidity, efficient packing abilities, and quality of active materials/electrolytes, play a crucial role. These factors directly affect the kinetics and reversibility of ions in batteries, which decides their overall electrochemical performance. Even commercially standardized samples encounter such challenges. To minimize this gap, theoretical studies should incorporate realistic battery operating conditions, and experimental testing should be conducted in sophisticated, controlled environments that closely align with theoretical predictions. In/ex situ characterization techniques can be employed to understand the typical structural and chemical changes during battery operations. Such observation can help to tune the material and to model the simulation methods. In addition, some standardized protocols can be established for theoretical as well as experimental studies, which can bridge the gap between theoretical and experimental observations.

## 7 Conclusions and Outlooks

The rapid discovery of different 2D layers for decades has paved the way for the development of numerous HR compositions, and their synergistic effect can substantially exhibit a spectrum of coupled redox reactions in batteries. Compared to conventional 2D counterparts, 2D HRs stand out as superior electrode materials owing to their fundamental physical and compositional changes that lead to improved conductivity, specific capacities, rate capability, diffusivity, and stability in monovalent batteries. Interestingly, selecting two different layered materials provides opportunities to assemble HRs with numerous configurations depending on the orientation of atoms and interaction between layers. This further leads to abundant research options in battery applications that focus on tuning the electrolyte/electrode interfacial properties and redox activity with M^+^ ions. Such remarkable outcomes have inspired the researchers to discover more profound insights into their application in batteries. Despite these outstanding results, the research of 2D HRs in batteries is still in its infancy, and it presents future scope for this area of research. Some critical future avenues for the researchers to exploit the full potential of 2D HRs are envisioned as follows:

1. Strategic selection of different families of 2D materials for HRs can help to ascertain novel battery chemistries. The redox potentials and number of electron transfers per charge–discharge cycle can be refined by ingeniously combining diverse 2D layers, thereby valuable in enhancing specific capacities. Furthermore, the interlayer interaction between different layers depends on their interfacial properties, including atomic/structural defects, functional group chemistries, porosity, morphology, and strain. These properties can be tuned to engineer HRs providing reduced interfacial resistance, enhanced charge transport, and mechanical stability. Defects and functionalities control the ion mobility and tailor the electrochemical activity. Likewise, the arrangement of layers can modulate internal strain, significantly impacting the overall structural integrity of HRs. Moreover, surface coatings can mitigate the degradation of the environmentally sensitive layered part of HRs. Together, these approaches present a wealth of exciting possibilities for future battery design and experimental exploration.
2. Developing a scalable and commercially viable synthesis method is a prerequisite for attaining these characteristics. Traditional techniques such as CVD, ALD, solution mixing, and hydro/solvothermal must be optimized for consistency, cost‐effectiveness, and throughput. Exploring roll‐to‐roll and printing‐compatible synthesis pathways can be helpful for the commercial translation of 2D HR‐based energy storage technologies.
3. The compatibility of electrodes (anode/cathode) and electrolytes is the fundamental factor deciding the performance of batteries. The availability of several classes of electrolytes, such as organic, solid‐state, aqueous, ionic liquids, and quasi‐solid‐state electrolytes, further provides an excellent opportunity for researchers to optimize the performance of 2D HRs in batteries. The remarkable structural properties of 2D HRs allow even large‐sized ions. The electrolyte chemistries corresponding to the desired M^+^ ion (Li^+^, Na^+^, K^+^, etc.) further widen the scope for innovations.
4. There is an ongoing discrepancy between the lab‐scale performances of half‐cells and commercial‐scale full‐cells. It is noteworthy that most of the HR materials have been tested only in half‐cell configurations. In general, the high‐capacity half‐cell performance of any material does not necessarily validate its versatility in full‐cell batteries. The redox potential, lattice structures, and surface interface energies are the most decisive factors ensuring the compatibility between anode and cathode materials. To develop a working prototype for the market, appropriate electrode materials, pre‐metallization, balanced weight loading, and electrolyte selection are the most prominent factors. These complexities open several challenges for researchers working in the theoretical and experimental domains.
5. For efficient commercial ESDs, it is necessary to look for their functioning in extreme conditions, including temperature (−20 to 60 °C), pressure (1000 psi or higher), and humidity (90%–100% relative humidity). Very little attention is paid to these conditions, leaving a broad room for future theoretical and experimental investigations.

We believe that the highlighted properties of 2D HR electrodes and electrolytes can be fine‐tuned to attain target‐oriented monovalent batteries that will benefit technological advances in the market.

## Conflict of Interest

The authors declare no conflict of interest.

## References

[1] Z. Huang , X. Li , Z. Chen , P. Li , X. Ji , C. Zhi , Nat. Rev. Chem. 2023, 7, 616. 37316580 10.1038/s41570-023-00506-w

[2] A. Innocenti , S. Beringer , S. Passerini , Nat. Rev. Mater. 2024, 9, 347.

[3] S. Li , K. Wang , G. Zhang , S. Li , Y. Xu , X. Zhang , X. Zhang , S. Zheng , X. Sun , Y. Ma , Adv. Funct. Mater. 2022, 32, 2200796.

[4] Z. He , Y. Huang , H. Liu , Z. Geng , Y. Li , S. Li , W. Deng , G. Zou , H. Hou , X. Ji , Nano Energy 2024, 129, 109996.

[5] W. Yan , Z. Mu , Z. Wang , Y. Huang , D. Wu , P. Lu , J. Lu , J. Xu , Y. Wu , T. Ma , M. Yang , X. Zhu , Y. Xia , S. Shi , L. Chen , H. Li , F. Wu , Nat. Energy 2023, 8, 800.

[6] C. Yan , R. Xu , Y. Xiao , J. Ding , L. Xu , B. Li , J. Huang , Adv. Funct. Mater. 2020, 30, 1909887.

[7] R. Wang , L. Wang , R. Liu , X. Li , Y. Wu , F. Ran , ACS Nano 2024, 18, 2611. 38221745 10.1021/acsnano.3c08712

[8] X. Li , Z. Huang , C. E. Shuck , G. Liang , Y. Gogotsi , C. Zhi , Nat. Rev. Chem. 2022, 6, 389. 37117426 10.1038/s41570-022-00384-8

[9] S. Mukherjee , G. Singh , ACS Appl. Energy Mater. 2019, 2, 932.

[10] J. Zheng , Y. Wu , Y. Sun , J. Rong , H. Li , L. Niu , Nano‐Micro Lett. 2021, 13, 12. 10.1007/s40820-020-00541-y PMC8187553 34138200

[11] S. Zhu , J. Li , X. Deng , C. He , E. Liu , F. He , C. Shi , N. Zhao , Adv. Funct. Mater. 2017, 27, 1605017.

[12] J. Zhu , G. Xiao , X. Zuo , Nano‐Micro Lett. 2020, 12, 120. 10.1007/s40820-020-00453-x PMC7770849 34138144

[13] J. Bi , Z. Du , J. Sun , Y. Liu , K. Wang , H. Du , W. Ai , W. Huang , Adv. Mater. 2023, 35, 2210734. 10.1002/adma.202210734 36623267

[14] H. Kaur , B. Konkena , M. McCrystall , K. Synnatschke , C. Gabbett , J. Munuera , R. Smith , Y. Jiang , R. Bekarevich , L. Jones , V. Nicolosi , J. N. Coleman , ACS Nano 2024, 18, 20213. 39038184 10.1021/acsnano.4c03501 PMC11308769

[15] C. Zhang , H. Pan , L. Sun , F. Xu , Y. Ouyang , F. Rosei , Energy Storage Mater. 2021, 38, 354.

[16] R. Rojaee , R. Shahbazian‐Yassar , ACS Nano 2020, 14, 2628. 32083832 10.1021/acsnano.9b08396

[17] Y. Li , J. Zhang , Q. Chen , X. Xia , M. Chen , Adv. Mater. 2021, 33, 2100855. 10.1002/adma.202100855 34033149

[18] D. Yuan , Y. Dou , Z. Wu , Y. Tian , K. H. Ye , Z. Lin , S. X. Dou , S. Zhang , Chem. Rev. 2022, 122, 957. 34709781 10.1021/acs.chemrev.1c00636

[19] E. Pomerantseva , Y. Gogotsi , Nat. Energy 2017, 2, 17089.

[20] H. Wang , Z. Cui , S. A. He , J. Zhu , W. Luo , Q. Liu , R. Zou , Nano‐Micro Lett. 2022, 14, 189. 10.1007/s40820-022-00935-0 PMC9482562 36114888

[21] Z. Zhang , P. Liu , Y. Song , Y. Hou , B. Xu , T. Liao , H. Zhang , J. Guo , Z. Sun , Adv. Sci. 2022, 9, 2204297. 10.1002/advs.202204297 PMC9762311 36266983

[22] J. Zhou , S. Zhao , F. Lv , H. Luo , S. Zhang , W. Zhang , F. Lin , W. Zhang , K. Wang , D. Wang , S. Guo , Adv. Funct. Mater. 2024, 34, 2409301.

[23] Q. Peng , Z. Wang , B. Sa , B. Wu , Z. Sun , ACS Appl. Mater. Interfaces 2016, 8, 13449. 27165567 10.1021/acsami.6b03368

[24] C. Tan , X. Cao , X. J. Wu , Q. He , J. Yang , X. Zhang , J. Chen , W. Zhao , S. Han , G. H. Nam , M. Sindoro , H. Zhang , Chem. Rev. 2017, 117, 6225. 28306244 10.1021/acs.chemrev.6b00558

[25] A. Castellanos‐Gomez , X. Duan , Z. Fei , H. R. Gutierrez , Y. Huang , X. Huang , J. Quereda , Q. Qian , E. Sutter , P. Sutter , Nat. Rev. Methods Primers 2022, 2, 58.

[26] H. W. Guo , Z. Hu , Z. B. Liu , J. G. Tian , Adv. Funct. Mater. 2021, 31, 2007810.

[27] J. Mei , T. Liao , Z. Sun , Energy Environ. Mater. 2022, 5, 115.

[28] X. Liu , M. C. Hersam , Adv. Mater. 2018, 30, 1801586. 10.1002/adma.201801586 30039558

[29] S. Liu , L. Kang , J. Henzie , J. Zhang , J. Ha , M. A. Amin , M. S. A. Hossain , S. C. Jun , Y. Yamauchi , ACS Nano 2021, 15, 18931. 34860483 10.1021/acsnano.1c08428

[30] M. Peng , K. Shin , L. Jiang , Y. Jin , K. Zeng , X. Zhou , Y. Tang , Angew. Chem., Int. Ed. 2022, 61, 2206770. 10.1002/anie.202206770 35689344

[31] I. Tantis , S. Talande , V. Tzitzios , G. Basina , V. Shrivastav , A. Bakandritsos , R. Zboril , Adv. Funct. Mater. 2023, 33, 2209360.

[32] S. H. Choi , S. J. Yun , Y. S. Won , C. S. Oh , S. M. Kim , K. K. Kim , Y. H. Lee , Nat. Commun. 2022, 13, 1484. 35304474 10.1038/s41467-022-29182-y PMC8933535

[33] A. Noori , M. F. El‐Kady , M. S. Rahmanifar , R. B. Kaner , M. F. Mousavi , Chem. Soc. Rev. 2019, 48, 1272. 30741286 10.1039/c8cs00581h

[34] K. Xu , Electrolytes, Interfaces and Interphases, Royal Society of Chemistry, London 2023.

[35] X. Wang , Q. Weng , Y. Yang , Y. Bando , D. Golberg , Chem. Soc. Rev. 2016, 45, 4042. 27196691 10.1039/c5cs00937e

[36] C. Heubner , M. Schneider , A. Michaelis , Adv. Energy Mater. 2020, 10, 1902523.

[37] Z. Chen , D. L. Danilov , R. A. Eichel , P. H. L. Notten , Adv. Energy Mater. 2022, 12, 2201506.

[38] K. Fu , X. Li , K. Sun , Z. Zhang , H. Yang , L. Gong , G. Qin , D. Hu , T. Li , P. Tan , Adv. Funct. Mater. 2024, 34, 2409623.

[39] X. Xu , F. Xu , X. Zhang , C. Qu , J. Zhang , Y. Qiu , R. Zhuang , H. Wang , Nano‐Micro Lett. 2022, 14, 91. 10.1007/s40820-022-00829-1 PMC8975989 35362824

[40] R. Andris , T. Averianov , M. J. Zachman , E. Pomerantseva , ACS Appl. Mater. Interfaces 2023, 15, 26525. 37216415 10.1021/acsami.2c22916

[41] K. Choudhary , K. F. Garrity , S. T. Hartman , G. Pilania , F. Tavazza , Phys. Rev. Mater. 2023, 7, 014009.

[42] Y. Lu , J. Chen , M. J. Coupin , S. Sinha , J. H. Warner , Adv. Mater. 2022, 34, 2205403. 10.1002/adma.202205403 36043938

[43] X. Yuan , Z. Zhang , Y. He , S. Zhao , N. Zhou , J. Phys. Chem. C 2022, 126, 91.

[44] X. Yang , B. Sa , P. Lin , C. Xu , Q. Zhu , H. Zhan , Z. Sun , J. Phys. Chem. C 2020, 124, 23699.

[45] G. Barik , S. Pal , J. Phys. Chem. C 2021, 125, 8980.

[46] D. X. Song , L. Xie , Y. F. Zhang , Y. Lu , M. An , W. G. Ma , X. Zhang , ACS Appl. Energy Mater. 2020, 3, 7699.

[47] C. Ye , Y. Jiao , H. Jin , A. D. Slattery , K. Davey , H. Wang , S. Qiao , Angew. Chem., Int. Ed. 2018, 57, 16703. 10.1002/anie.201810579 30325094

[48] J. Zhang , W. Xie , X. Xu , S. Zhang , J. Zhao , Chem. Mater. 2016, 28, 5022.

[49] R. Sen , K. Jatkar , P. Johari , Phys. Rev. B 2020, 101, 235425.

[50] J. Yu , M. Zhou , M. Yang , Q. Yang , Z. Zhang , Y. Zhang , ACS Appl. Energy Mater. 2020, 3, 11699.

[51] H. Xie , B. Chen , C. Liu , G. Wu , S. Sui , E. Liu , G. Zhou , C. He , W. Hu , N. Zhao , Energy Storage Mater. 2023, 60, 102830.

[52] M. R. Busche , T. Drossel , T. Leichtweiss , D. A. Weber , M. Falk , M. Schneider , M. L. Reich , H. Sommer , P. Adelhelm , J. Janek , Nat. Chem. 2016, 8, 426. 27102676 10.1038/nchem.2470

[53] A. Chaves , J. G. Azadani , H. Alsalman , D. R. da Costa , R. Frisenda , A. J. Chaves , S. H. Song , Y. D. Kim , D. He , J. Zhou , A. Castellanos‐Gomez , F. M. Peeters , Z. Liu , C. L. Hinkle , S. H. Oh , P. D. Ye , S. J. Koester , Y. H. Lee , P. Avouris , X. Wang , T. Low , npj 2D Mater. Appl. 2020, 4, 29.

[54] M. Zhao , N. Trainor , C. E. Ren , M. Torelli , B. Anasori , Y. Gogotsi , Adv. Mater. Technol. 2019, 4, 1800639.

[55] A. K. Nair , C. M. Da Silva , C. H. Amon , J. Phys. Chem. C 2023, 127, 9541.

[56] T. Roy , M. Tosun , M. Hettick , G. H. Ahn , C. Hu , A. Javey , Appl. Phys. Lett. 2016, 108, 083111.

[57] H. Chen , R. Liu , Y. Wu , J. Cao , J. Chen , Y. Hou , Y. Guo , R. Khatoon , L. Chen , Q. Zhang , Q. He , J. Lu , Chem. Eng. J. 2021, 407, 126973.

[58] M. Okada , A. Kutana , Y. Kureishi , Y. Kobayashi , Y. Saito , T. Saito , K. Watanabe , T. Taniguchi , S. Gupta , Y. Miyata , B. I. Yakobson , H. Shinohara , R. Kitaura , ACS Nano 2018, 12, 2498. 29481065 10.1021/acsnano.7b08253

[59] X. Wang , L. Chen , Y. Yu , W. Wang , L. Yue , Z. Shao , H. Wu , Y. Li , Adv. Funct. Mater. 2024, 34, 2406290.

[60] D. Lu , X. Wang , Y. Hu , L. Yue , Z. Shao , W. Zhou , L. Chen , W. Wang , Y. Li , Adv. Funct. Mater. 2023, 33, 2212689.

[61] Y. Liu , T. Zhou , Y. Zheng , Z. He , C. Xiao , W. K. Pang , W. Tong , Y. Zou , B. Pan , Z. Guo , Y. Xie , ACS Nano 2017, 11, 8519. 28745871 10.1021/acsnano.7b04617

[62] L. Pan , R. Hu , Y. Zhang , D. Sha , X. Cao , Z. Li , Y. Zhao , J. Ding , Y. Wang , Z. Sun , Nano‐Micro Lett. 2023, 15, 225. 10.1007/s40820-023-01202-6 PMC10575839 37831299

[63] T. Wang , M. Li , L. Qi , P. Jie , W. Yang , Y. Li , Adv. Funct. Mater. 2023, 33, 2308470.

[64] J. Ni , M. Sun , L. Li , Adv. Mater. 2019, 31, 1902603. 10.1002/adma.201902603 31465132

[65] X. Lu , Y. Shi , D. Tang , X. Lu , Z. Wang , N. Sakai , Y. Ebina , T. Taniguchi , R. Ma , T. Sasaki , C. Yan , ACS Nano 2022, 16, 4775. 35235304 10.1021/acsnano.2c00089

[66] L. Zhong , M. Yue , Y. Liang , B. Xi , X. An , Y. Xiao , B. Cheng , S. Lei , S. Xiong , Adv. Funct. Mater. 2024, 34, 2407740.

[67] W. Wang , X. Wang , L. Chen , D. Lu , W. Zhou , Y. Li , Chem. Eng. J. 2023, 461, 142100.

[68] Z. Zhu , Q. Lv , Y. Ni , S. Gao , J. Geng , J. Liang , F. Li , Angew. Chem., Int. Ed. 2022, 61, 2116699. 10.1002/anie.202116699 35018699

[69] J. Fan , H. Chen , X. Niu , Appl. Phys. Lett. 2024, 125, 143901.

[70] R. Zhao , Z. Qian , Z. Liu , D. Zhao , X. Hui , G. Jiang , C. Wang , L. Yin , Nano Energy 2019, 65, 104037.

[71] J. Wang , Z. Li , Q. Wang , H. Sun , H. J. Woo , S. B. Aziz , N. Z. N. Husin , R. T. Subramaniam , B. Wang , ACS Mater. Lett. 2024, 6, 222.

[72] X. Shi , J. Li , X. Zhang , M. Li , Q. Jing , G. Fang , M. Long , J. Phys. Chem. C 2024, 128, 6189.

[73] C. Zhang , F. Han , F. Wang , Q. Liu , D. Zhou , F. Zhang , S. Xu , C. Fan , X. Li , J. Liu , Energy Storage Mater. 2020, 24, 208.

[74] W. Yu , B. Cui , J. Han , S. Zhu , X. Xu , J. Tan , Q. Xu , Y. Min , Y. Peng , H. Liu , Y. Wang , Adv. Sci. 2024, 11, 2405135. 10.1002/advs.202405135 PMC11423093 39049722

[75] Q. Li , J. Yang , L. Zhang , J. Phys. Chem. C 2018, 122, 18294.

[76] P. Xiang , X. Chen , J. Liu , B. Xiao , L. Yang , J. Phys. Chem. C 2018, 122, 9302.

[77] X. Guo , W. Zhang , J. Zhang , D. Zhou , X. Tang , X. Xu , B. Li , H. Liu , G. Wang , ACS Nano 2020, 14, 3651. 32150388 10.1021/acsnano.0c00177

[78] J. Li , Q. Peng , J. Zhou , Z. Sun , J. Phys. Chem. C 2019, 123, 11493.

[79] K. Yuan , P. Hao , Y. Zhou , X. Hu , J. Zhang , S. Zhong , Phys. Chem. Chem. Phys. 2022, 24, 13713. 35612407 10.1039/d1cp05707c

[80] Y. Chen , Q. Wang , Q. Zhang , S. Zhang , Y. Zhang , Phys. Chem. Chem. Phys. 2023, 25, 26557. 37753582 10.1039/d3cp03295g

[81] D. Wang , L. M. Liu , S. J. Zhao , Z. Y. Hu , H. Liu , J. Phys. Chem. C 2016, 120, 4779.

[82] X. Wu , H. Wang , Z. Zhao , B. Huang , J. Mater. Chem. A 2020, 8, 12705.

[83] Y. T. Du , X. Kan , F. Yang , L. Y. Gan , U. Schwingenschlögl , ACS Appl. Mater. Interfaces 2018, 10, 32867. 30160474 10.1021/acsami.8b10729

[84] S. Mukherjee , L. Kavalsky , C. V. Singh , ACS Appl. Mater. Interfaces 2018, 10, 8630. 29436225 10.1021/acsami.7b18595

[85] M. Ai , J. Sun , Z. Li , H. Liang , C. Liu , J. Phys. Chem. C 2021, 125, 11391.

[86] Z. Y. Song , Y. D. Cao , L. L. Fan , J. Song , Y. Feng , H. Liu , C. L. Lv , G. G. Gao , Rare Met. 2024, 44, 195.

[87] J. Zhang , Y. F. Zhang , S. P. Huang , W. Lin , W. K. Chen , J. Phys. Chem. C 2019, 123, 30809.

[88] C. He , J. H. Zhang , W. X. Zhang , T. T. Li , J. Phys. Chem. C 2019, 123, 5157.

[89] H. Liu , Z. Huang , G. Wu , Y. Wu , G. Yuan , C. He , X. Qi , J. Zhong , J. Mater. Chem. A 2018, 6, 17040.

[90] K. Fan , J. Tang , S. Wu , C. Yang , J. Hao , Phys. Chem. Chem. Phys. 2017, 19, 267. 10.1039/c6cp05983j 27901140

[91] M. Ma , S. Zhang , Y. Yao , H. Wang , H. Huang , R. Xu , J. Wang , X. Zhou , W. Yang , Z. Peng , X. Wu , Y. Hou , Y. Yu , Adv. Mater. 2020, 32, 2000958. 10.1002/adma.202000958 32323393

[92] W. Chen , K. Hu , H. Zheng , Y. Pan , Z. Lv , X. Tu , C. Zheng , T. He , F. Huang , W. Dong , Small 2024, 20, 2311638. 10.1002/smll.202311638 38342598

[93] T. Wang , K. Yao , Y. Hua , E. G. Shankar , R. Shanthappa , J. S. Yu , Chem. Eng. J. 2023, 457, 141363.

[94] E. Xu , Y. Zhang , H. Wang , Z. Zhu , J. Quan , Y. Chang , P. Li , D. Yu , Y. Jiang , Chem. Eng. J. 2020, 385, 123839.

[95] Y. Aierken , C. Sevik , O. Gülseren , F. M. Peeters , D. Çakır , J. Mater. Chem. A 2018, 6, 2337.

[96] L. Fang , Z. Lan , W. Guan , P. Zhou , N. Bahlawane , W. Sun , Y. Lu , C. Liang , M. Yan , Y. Jiang , Energy Storage Mater. 2019, 18, 107.

[97] S. Faramarzi , T. Movlarooy , ACS Appl. Mater. Interfaces 2024, 16, 25966. 38742729 10.1021/acsami.3c17997

[98] D. Zhu , Q. Zhang , X. Li , Y. Zhang , J. Phys. Chem. C 2021, 125, 4391.

[99] Q. Pan , Z. Tong , Y. Su , Y. Zheng , L. Shang , Y. Tang , Adv. Mater. 2022, 34, 2203485. 10.1002/adma.202203485 35962631

[100] W. Liu , X. Zhang , Y. Xu , L. Wang , Z. Li , C. Li , K. Wang , X. Sun , Y. An , Z. Wu , Y. Ma , Adv. Funct. Mater. 2022, 32, 2202342.

[101] J. Yang , J. Luo , Y. Kuang , Y. He , P. Wen , L. Xiong , X. Wang , Z. Yang , ACS Appl. Mater. Interfaces 2021, 13, 2072. 33347756 10.1021/acsami.0c19934

[102] B. Liu , T. Gao , P. Liao , Y. Wen , M. Yao , S. Shi , W. Zhang , Phys. Chem. Chem. Phys. 2021, 23, 18784. 34612417 10.1039/d1cp02243a

[103] D. Guo , F. Ming , D. B. Shinde , L. Cao , G. Huang , C. Li , Z. Li , Y. Yuan , M. N. Hedhili , H. N. Alshareef , Z. Lai , Adv. Funct. Mater. 2021, 31, 2101194.

[104] T. Wang , D. Legut , Y. Fan , J. Qin , X. Li , Q. Zhang , Nano Lett. 2020, 20, 6199. 32787187 10.1021/acs.nanolett.0c02595

[105] R. Li , W. Li , A. Singh , D. Ren , Z. Hou , M. Ouyang , Energy Storage Mater. 2022, 52, 395.

[106] H. Li , T. Yamaguchi , S. Matsumoto , H. Hoshikawa , T. Kumagai , N. L. Okamoto , T. Ichitsubo , Nat. Commun. 2020, 11, 1584. 32284535 10.1038/s41467-020-15452-0 PMC7154030

[107] L. Li , J. Ren , J. Li , X. Guo , M. Liu , X. Lu , J. Mater. Chem. C 2023, 11, 14151.

[108] J. Su , W. Li , T. Duan , B. Xiao , X. Wang , Y. Pei , X. C. Zeng , Carbon 2019, 153, 767.

[109] F. M. Ma , X. M. Zhao , H. B. Luo , C. L. Shang , H. M. Gao , X. L. Wang , J. Mater. Chem. A 2024, 12, 23008.

[110] X. Zou , Y. Huang , Y. Chen , C. Cai , M. Qiu , Y. Zhang , J. Zhu , Appl. Surf. Sci. 2023, 614, 156169.

[111] M. You , M. Zhang , G. Guo , S. Luo , J. Zhong , Electrochim. Acta 2023, 463, 142799.

[112] G. Barik , S. Pal , Phys. Chem. Chem. Phys. 2020, 22, 1701. 31895351 10.1039/c9cp04349g

[113] A. Xu , Q. Zhu , G. Li , C. Gong , X. Li , H. Chen , J. Cui , S. Wu , Z. Xu , Y. Yan , Small 2022, 18, 2203976. 10.1002/smll.202203976 36089671

[114] M. Zhang , K. Huang , Y. Zou , J. Jia , L. Wu , W. Zeng , Chem. Eng. J. 2024, 499, 156547.

[115] M. Ma , S. Zhang , L. Wang , Y. Yao , R. Shao , L. Shen , L. Yu , J. Dai , Y. Jiang , X. Cheng , Y. Wu , X. Wu , X. Yao , Q. Zhang , Y. Yu , Adv. Mater. 2021, 33, 2106232. 10.1002/adma.202106232 34558122

[116] G. Barik , S. Pal , Phys. Chem. Chem. Phys. 2024, 26, 18054. 38895793 10.1039/d4cp00940a

[117] K. Zhang , M. Pan , Y. Wang , X. Wang , W. Sun , Mater. Today Commun. 2024, 40, 109779.

[118] W. Zhang , Y. Wu , Z. Xu , H. Li , M. Xu , J. Li , Y. Dai , W. Zong , R. Chen , L. He , Z. Zhang , D. J. L. Brett , G. He , Y. Lai , I. P. Parkin , Adv. Energy Mater. 2022, 12, 2201065.

[119] Z. Bo , Z. Zheng , Y. Huang , P. Chen , J. Yan , K. Cen , R. Mo , H. Yang , K. (Ken) Ostrikov , Chem. Eng. J. 2024, 485, 149837.

[120] R. Chen , Y. Zhou , J. He , X. Li , Adv. Funct. Mater. 2024, 34, 2407986.

[121] I. Demiroglu , F. M. Peeters , O. Gulseren , D. Cakır , C. Sevik , J. Phys. Chem. Lett. 2019, 10, 727. 30694678 10.1021/acs.jpclett.8b03056

[122] D. K. Bediako , M. Rezaee , H. Yoo , D. T. Larson , S. Y. F. Zhao , T. Taniguchi , K. Watanabe , T. L. Brower‐Thomas , E. Kaxiras , P. Kim , Nature 2018, 558, 425. 29925970 10.1038/s41586-018-0205-0

[123] G. Barik , S. Pal , Appl. Surf. Sci. 2022, 596, 153529.

[124] P. V. Pham , S. C. Bodepudi , K. Shehzad , Y. Liu , Y. Xu , B. Yu , X. Duan , Chem. Rev. 2022, 122, 6514. 35133801 10.1021/acs.chemrev.1c00735

[125] X. Liu , J. Pei , Z. Hu , W. Zhao , S. Liu , M. R. Amara , K. Watanabe , T. Taniguchi , H. Zhang , Q. Xiong , Nano Lett. 2020, 20, 5359. 32543201 10.1021/acs.nanolett.0c01722

[126] H. Rokni , W. Lu , Nat. Commun. 2020, 11, 5607. 33154376 10.1038/s41467-020-19411-7 PMC7645779

[127] S. Chen , G. Chen , Y. Zhao , S. Bu , Z. Hu , B. Mao , H. Wu , J. Liao , F. Li , C. Zhou , B. Guo , W. Liu , Y. Zhu , Q. Lu , J. Hu , M. Shang , Z. Shi , B. Yu , X. Zhang , Z. Zhao , K. Jia , Y. Zhang , P. Sun , Z. Liu , L. Lin , X. Wang , Adv. Mater. 2024, 36, 2308950. 10.1002/adma.202308950 38288661

[128] T. F. Schranghamer , M. Sharma , R. Singh , S. Das , Chem. Soc. Rev. 2021, 50, 11032. 34397050 10.1039/d1cs00706h

[129] Y. Zhang , Y. Yao , M. G. Sendeku , L. Yin , X. Zhan , F. Wang , Z. Wang , J. He , Adv. Mater. 2019, 31, 1901694. 10.1002/adma.201901694 31402526

[130] E. Lee , S. G. Lee , W. H. Lee , H. C. Lee , N. N. Nguyen , M. S. Yoo , K. Cho , Chem. Mater. 2020, 32, 4544.

[131] R. Zhang , M. Li , L. Li , Z. Wei , F. Jiao , D. Geng , W. Hu , Adv. Funct. Mater. 2021, 31, 2102049.

[132] Z. Cai , B. Liu , X. Zou , H. M. Cheng , Chem. Rev. 2018, 118, 6091. 29384374 10.1021/acs.chemrev.7b00536

[133] X. X. Yu , L. Wang , H. Yin , Appl. Mater. Today 2019, 15, 582.

[134] H. Ci , J. Cai , H. Ma , Z. Shi , G. Cui , M. Wang , J. Jin , N. Wei , C. Lu , W. Zhao , J. Sun , Z. Liu , ACS Nano 2020, 14, 11929. 32790327 10.1021/acsnano.0c05030

[135] N. Bansal , K. P. Agrim , A. M. Bohra , T. Ahamad , C. Park , H. Ahn , R. R. Salunkhe , ACS Appl. Nano Mater. 2024, 7, 17305.

[136] Y. Jin , H. Yu , X. Liang , Appl. Phys. Rev. 2021, 8, 031301.

[137] X. Wang , G. Yushin , Energy Environ. Sci. 2015, 8, 1889.

[138] C. Zhou , M. Li , N. Hu , J. Yang , H. Li , J. Yan , P. Lei , Y. Zhuang , S. Guo , Adv. Funct. Mater. 2022, 32, 2204635.

[139] B. Gupta , M. A. Hossain , A. Riaz , A. Sharma , D. Zhang , H. H. Tan , C. Jagadish , K. Catchpole , B. Hoex , S. Karuturi , Adv. Funct. Mater. 2022, 32, 2109105.

[140] K. P. Musselman , C. F. Uzoma , M. S. Miller , Chem. Mater. 2016, 28, 8443.

[141] Y. Ren , Q. Zhai , B. Wang , L. Hu , Y. Ma , Y. Dai , S. Tang , X. Meng , Chem. Eng. J. 2022, 439, 135535.

[142] Y. Chao , R. Jalili , Y. Ge , C. Wang , T. Zheng , K. Shu , G. G. Wallace , Adv. Funct. Mater. 2017, 27, 1700234.

[143] Y. Wang , J. Song , W. Wong , Angew. Chem. 2023, 135, 2218343.

[144] J. Zhou , T. Wu , Y. Pan , J. Zhu , X. Chen , C. Peng , C. Shu , L. Kong , W. Tang , S. Chou , Adv. Funct. Mater. 2022, 32, 2106966.

[145] S. Tang , Q. Yuan , J. Wang , T. Wang , W. Xiang , J. Li , J. S. Yu , Energy Storage Mater. 2024, 68, 103357.

[146] X. Ou , Z. Xiao , J. Zhang , C. Wang , D. Wang , B. Zhang , Y. Wu , ACS Nano 2020, 14, 13952. 32941006 10.1021/acsnano.0c06371

[147] C. Chen , X. Xie , B. Anasori , A. Sarycheva , T. Makaryan , M. Zhao , P. Urbankowski , L. Miao , J. Jiang , Y. Gogotsi , Angew. Chem., Int. Ed. 2018, 57, 1846. 10.1002/anie.201710616 29292844

[148] M. Wang , X. Liu , B. Qin , Z. Li , Y. Zhang , W. Yang , H. Fan , Chem. Eng. J. 2023, 451, 138508.

[149] S. Cheng , Z. Zuo , Y. Li , Mater. Chem. Front. 2024, 8, 1835.

[150] J. Liu , Y. Chang , K. Sun , P. Guo , D. Cao , Y. Ma , D. Liu , Q. Liu , Y. Fu , J. Liu , D. He , ACS Appl. Mater. Interfaces 2022, 14, 11739. 35200005 10.1021/acsami.1c18268

[151] J. Mei , J. Shang , C. Zhang , D. Qi , L. Kou , B. Wijerathne , C. Hu , T. Liao , J. MacLeod , Z. Sun , Small Methods 2022, 6, 2200658. 10.1002/smtd.202200658 35802910

[152] D. Zhang , T. Liu , J. Cheng , Q. Cao , G. Zheng , S. Liang , H. Wang , M. S. Cao , Nano‐Micro Lett. 2019, 11, 38. 10.1007/s40820-019-0270-4 PMC7770953 34137981

[153] Y. Lv , H. Pan , J. Lin , Z. Chen , Y. Li , H. Li , M. Shi , R. Yin , S. Zhu , Chem. Eng. J. 2022, 428, 132072.

[154] Z. Liu , H. Lv , Y. Xie , J. Wang , J. Fan , B. Sun , L. Jiang , Y. Zhang , R. Wang , K. Shi , J. Mater. Chem. A 2022, 10, 11980.

[155] H. Dai , X. Zhao , H. Xu , J. Yang , J. Zhou , Q. Chen , G. Sun , ACS Nano 2022, 16, 5556. 35426659 10.1021/acsnano.1c10212

[156] R. Fan , C. Zhao , J. Ma , S. Lei , G. Liang , T. He , G. Zhu , Y. Cai , J. Mater. Chem. A 2022, 10, 939.

[157] X. Wang , Q. Chen , C. Shen , J. Dai , C. Zhu , J. Zhang , Z. Wang , Q. Song , L. Wang , H. Li , Q. Wang , Z. Liu , Z. Luo , X. Huang , W. Huang , ACS Nano 2021, 15, 12171. 34269058 10.1021/acsnano.1c03688

[158] S. Wu , W. Wang , J. Shan , X. Wang , D. Lu , J. Zhu , Z. Liu , L. Yue , Y. Li , Energy Storage Mater. 2022, 49, 153.

[159] Q. H. Nguyen , H. Kim , I. T. Kim , W. Choi , J. Hur , Chem. Eng. J. 2020, 382, 122981.

[160] C. Deng , Y. Gao , Y. Yao , B. Liang , S. Lu , T. Tao , J. Mater. Chem. A 2022, 10, 11766.

[161] L. Wang , D. Liu , L. Jiang , Y. Ma , G. Yang , Y. Qian , W. Lei , Nano Energy 2022, 98, 107192.

[162] K. Bai , J. C. Fan , P. H. Shi , Y. L. Min , Q. J. Xu , J. Power Sources 2020, 456, 228003.

[163] S. Li , Y. Wang , C. Lai , J. Qiu , M. Ling , W. Martens , H. Zhao , S. Zhang , J. Mater. Chem. A 2014, 2, 10211.

[164] J. Yan , C. E. Ren , K. Maleski , C. B. Hatter , B. Anasori , P. Urbankowski , A. Sarycheva , Y. Gogotsi , Adv. Funct. Mater. 2017, 27, 1701264.

[165] C. Wei , L. Tan , Y. Zhang , B. Xi , S. Xiong , J. Feng , ACS Appl. Mater. Interfaces 2022, 14, 2979. 34995069 10.1021/acsami.1c22787

[166] W. J. Ong , L. L. Tan , S. P. Chai , S. T. Yong , A. R. Mohamed , Nano Energy 2015, 13, 757.

[167] W. Tian , A. VahidMohammadi , Z. Wang , L. Ouyang , M. Beidaghi , M. M. Hamedi , Nat. Commun. 2019, 10, 2558. 31186411 10.1038/s41467-019-10631-0 PMC6560128

[168] J. Zeng , J. Huang , J. Liu , T. Xie , C. Peng , Y. Lu , P. Lu , R. Zhang , J. Min , Carbon 2019, 154, 24.

[169] M. Cai , J. Yang , X. Lu , X. Lu , ACS Appl. Nano Mater. 2024, 7, 27940.

[170] P. Xiong , R. Ma , N. Sakai , T. Sasaki , ACS Nano 2018, 12, 1768. 29355303 10.1021/acsnano.7b08522

[171] P. Xiong , R. Ma , N. Sakai , L. Nurdiwijayanto , T. Sasaki , ACS Energy Lett. 2018, 3, 997.

[172] J. Jang , H. J. Jung , S. Chong , D. Kim , J. Kim , S. O. Kim , I. Kim , Adv. Mater. 2020, 32, 2002723. 10.1002/adma.202002723 32700344

[173] J. Wang , J. Tang , B. Ding , V. Malgras , Z. Chang , X. Hao , Y. Wang , H. Dou , X. Zhang , Y. Yamauchi , Nat. Commun. 2017, 8, 15717. 28604671 10.1038/ncomms15717 PMC5472787

[174] N. Kumar , N. Bansal , Y. Yamauchi , R. R. Salunkhe , Chem. Mater. 2022, 34, 4946.

[175] H. Shi , J. Qin , K. Huang , P. Lu , C. (John) Zhang , Y. Dong , M. Ye , Z. Liu , Z. S. Wu , Angew. Chem., Int. Ed. 2020, 59, 12147. 10.1002/anie.202004284 32237031

[176] N. Kumar , N. Bansal , R. R. Salunkhe , Chem. Commun. 2021, 57, 13748. 10.1039/d1cc05978e 34852029

[177] S. Kim , M. Ju , J. Lee , J. Hwang , J. Lee , J. Am. Chem. Soc. 2020, 142, 9250. 32053749 10.1021/jacs.0c00311

[178] K. Lan , Q. Wei , R. Wang , Y. Xia , S. Tan , Y. Wang , A. Elzatahry , P. Feng , L. Mai , D. Zhao , J. Am. Chem. Soc. 2019, 141, 16755. 31564098 10.1021/jacs.9b06962

[179] Z. Liu , R. Zhang , H. Xiong , L. Zhang , J. Li , L. Wang , Z. Qiao , Adv. Mater. Interfaces 2023, 10, 2202501.

[180] C. Chen , S. Zhang , B. Huang , Y. Peng , Y. Zhang , L. Wang , Y. Wang , Adv. Funct. Mater. 2024, 34, 2410248.

[181] J. Wang , V. Malgras , Y. Sugahara , Y. Yamauchi , Nat. Commun. 2021, 12, 3563. 34117228 10.1038/s41467-021-23819-0 PMC8196154

[182] Y. Fang , Y. Lv , F. Gong , A. A. Elzatahry , G. Zheng , D. Zhao , Adv. Mater. 2016, 28, 9385. 27601056 10.1002/adma.201602210

[183] J. Wang , Z. Chang , B. Ding , T. Li , G. Yang , Z. Pang , T. Nakato , M. Eguchi , Y. Kang , J. Na , B. Y. Guan , Y. Yamauchi , Angew. Chem., Int. Ed. 2020, 59, 19570. 10.1002/anie.202007063 32652751

[184] X. Xi , D. Wu , L. Han , Y. Yu , Y. Su , W. Tang , R. Liu , ACS Nano 2018, 12, 5436. 29733630 10.1021/acsnano.8b00576

[185] S. Liu , P. Gordiichuk , Z. S. Wu , Z. Liu , W. Wei , M. Wagner , N. Mohamed‐Noriega , D. Wu , Y. Mai , A. Herrmann , K. Mullen , X. Feng , Nat. Commun. 2015, 6, 8817. 26577914 10.1038/ncomms9817 PMC4660032

[186] S. Masubuchi , M. Morimoto , S. Morikawa , M. Onodera , Y. Asakawa , K. Watanabe , T. Taniguchi , T. Machida , Nat. Commun. 2018, 9, 1413. 29650955 10.1038/s41467-018-03723-w PMC5897399

[187] A. D. Refino , C. Eldona , R. F. H. Hernandha , E. Adhitama , A. Sumboja , E. Peiner , H. S. Wasisto , Commun. Mater. 2024, 5, 22.

[188] P. Gao , L. Wang , Y. Y. Zhang , Y. Huang , L. Liao , P. Sutter , K. Liu , D. Yu , E. G. Wang , Nano Lett. 2016, 16, 5582. 27504584 10.1021/acs.nanolett.6b02136

[189] J. Ding , H. Li , S. Wang , S. Wu , L. Zhang , L. Zhou , S. Fang , Y. Yu , Nano Energy 2024, 129, 110042.

[190] W. Cao , J. Zhang , H. Li , Energy Storage Mater. 2020, 26, 46.

[191] Y. Xiao , Y. Miao , F. Gong , T. Zhang , L. Zhou , Q. Yu , S. Hu , S. Chen , Small 2024, 20, 2311703. 10.1002/smll.202311703 38459649

[192] S. Kim , J. Hwang , Y. Jo , C. Park , N. Bansal , R. R. Salunkhe , H. Ahn , J. Mater. Chem. A 2024, 12, 16143.

[193] K. V. Kravchyk , M. V. Kovalenko , ACS Energy Lett. 2023, 8, 1266. 10.1021/acsenergylett.5c03429 PMC12803501 41541646

[194] X. Dong , Z. Li , D. Luo , K. Huang , H. Dou , X. Zhang , Adv. Funct. Mater. 2023, 33, 2210473.

[195] T. Wang , J. Zhao , L. Qi , G. Li , W. Yang , Y. Li , Energy Storage Mater. 2023, 54, 10.

[196] X. Wang , S. Zhang , Y. Shan , L. Chen , G. Gao , X. Zhu , B. Cao , X. He , Energy Storage Mater. 2021, 37, 55.

[197] J. Quinn , B. Wu , Y. Xu , M. H. Engelhard , J. Xiao , C. Wang , ACS Nano 2022, 16, 21063. 36520937 10.1021/acsnano.2c08777

[198] Z. W. Yin , W. Zhao , J. Li , X. X. Peng , C. Lin , M. Zhang , Z. Zeng , H. G. Liao , H. Chen , H. Lin , F. Pan , Adv. Funct. Mater. 2022, 32, 2107190.

[199] J. Cai , Y. Zhou , S. Tao , Y. Liu , W. Deng , H. Hou , G. Zou , X. Ji , Energy Storage Mater. 2024, 71, 103582.

[200] P. Gao , Z. Chen , Y. Gong , R. Zhang , H. Liu , P. Tang , X. Chen , S. Passerini , J. Liu , Adv. Energy Mater. 2020, 10, 1903780.

[201] P. Yan , L. Ji , X. Liu , Q. Guan , J. Guo , Y. Shen , H. Zhang , W. Wei , X. Cui , Q. Xu , Nano Energy 2021, 86, 106139.

[202] L. Seidl , N. Bucher , E. Chu , S. Hartung , S. Martens , O. Schneider , U. Stimming , Energy Environ. Sci. 2017, 10, 1631.

[203] O. Deniz , C. Sanchez‐Sanchez , T. Dumslaff , X. Feng , A. Narita , K. Müllen , N. Kharche , V. Meunier , R. Fasel , P. Ruffieux , Nano Lett. 2017, 17, 2197. 28301723 10.1021/acs.nanolett.6b04727

[204] S. S. Mai , K. Y. Hsiao , Y. C. Yang , Y. R. Lu , M. Y. Lu , Y. Y. Hsieh , C. B. Chang , H. Y. Tuan , Chem. Eng. J. 2023, 474, 145992.

[205] H. Wu , L. Wei , W. Li , C. Shi , X. Yao , Q. Fu , H. Li , X. Guo , Adv. Funct. Mater. 2024, 34, 2403729.

[206] K. Zhang , F. Wu , X. Wang , L. Zheng , X. Yang , H. Zhao , Y. Sun , W. Zhao , Y. Bai , C. Wu , Adv. Funct. Mater. 2022, 32, 2107764.

[207] M. Q. Zhao , M. Torelli , C. E. Ren , M. Ghidiu , Z. Ling , B. Anasori , M. W. Barsoum , Y. Gogotsi , Nano Energy 2016, 30, 603.

[208] T. Wang , M. Li , L. Yao , W. Yang , Y. Li , Adv. Mater. 2024, 36, 2402961. 10.1002/adma.202402961 38727517

[209] X. Zhu , H. Dong , Y. Liu , Y. H. Feng , Y. Tang , L. Yu , S. W. Xu , G. X. Wei , S. Sun , M. Liu , B. Xiao , R. Xu , Y. Xiao , S. Chou , P. F. Wang , ACS Nano 2024, 18, 32003. 39523605 10.1021/acsnano.4c09918

[210] S. You , Q. Zhang , J. Liu , Q. Deng , Z. Sun , D. Cao , T. Liu , K. Amine , C. Yang , Energy Environ. Sci. 2024, 17, 8189.

[211] P. Ge , M. Fouletier , Solid State Ionics 1172, 1988, 28.

[212] L. Zhang , W. A. Wang , S. Lu , Y. Xiang , Adv. Energy Mater. 2021, 11, 2003640.

[213] C. Wang , J. Xu , S. Shi , Y. Zhang , Z. Liu , X. Zhang , S. Yin , L. Li , RSC Adv. 2016, 6, 4422.

[214] Z. Xu , J. Wang , Adv. Energy Mater. 2022, 12, 2201692.

[215] E. Olsson , J. Yu , H. Zhang , H. M. Cheng , Q. Cai , Adv. Energy Mater. 2022, 12, 2200662.

[216] B. Jache , P. Adelhelm , Angew. Chem., Int. Ed. 2014, 53, 10169. 10.1002/anie.201403734 25056756

[217] X. Kong , X. Zhao , C. Li , Z. Jia , C. Yang , Z. Wu , X. Zhao , Y. Zhao , F. He , Y. Ren , P. Yang , Z. Liu , Small 2023, 19, 2206563. 10.1002/smll.202206563 36642823

[218] X. Ou , L. Cao , X. Liang , F. Zheng , H. S. Zheng , X. Yang , J. H. Wang , C. Yang , M. Liu , ACS Nano 2019, 13, 3666. 30785716 10.1021/acsnano.9b00375

[219] Y. Yi , Z. Sun , C. Li , Z. Tian , C. Lu , Y. Shao , J. Li , J. Sun , Z. Liu , Adv. Funct. Mater. 2020, 30, 1903878.

[220] Y. Cui , L. Zhao , B. Li , W. Feng , T. Cai , X. Li , H. Wang , D. Kong , Z. Fan , L. Zhi , Z. Yan , Q. Xue , W. Xing , Chem. Eng. J. 2022, 450, 137815.

[221] J. Cao , J. Li , D. Li , Z. Yuan , Y. Zhang , V. Shulga , Z. Sun , W. Han , Nano‐Micro Lett. 2021, 13, 113. 10.1007/s40820-021-00623-5 PMC8062676 34138334

[222] Z. Xia , X. Chen , H. Ci , Z. Fan , Y. Yi , W. Yin , N. Wei , J. Cai , Y. Zhang , J. Sun , J. Energy Chem. 2021, 53, 155.

[223] L. Cao , B. Luo , B. Xu , J. Zhang , C. Wang , Z. Xiao , S. Li , Y. Li , B. Zhang , G. Zou , H. Hou , X. Ou , X. Ji , Adv. Funct. Mater. 2021, 31, 2103802.

[224] H. He , D. Huang , Q. Gan , J. Hao , S. Liu , Z. Wu , W. K. Pang , B. Johannessen , Y. Tang , J. L. Luo , H. Wang , Z. Guo , ACS Nano 2019, 13, 11843. 31545592 10.1021/acsnano.9b05865

[225] B. Luo , P. Wu , J. Zhang , L. Cao , C. Wang , B. Lu , B. Zhang , X. Ou , Nano Res. 2021, 14, 3854.

[226] F. Chen , D. Shi , M. Yang , H. Jiang , Y. Shao , S. Wang , B. Zhang , J. Shen , Y. Wu , X. Hao , Adv. Funct. Mater. 2021, 31, 2007132.

[227] B. Liu , H. Zhang , C. Yuan , Q. Geng , Y. Li , J. Hu , Z. Lu , J. Xie , A. Hao , Y. Cao , J. Colloid Interface Sci. 2023, 646, 34. 37182257 10.1016/j.jcis.2023.05.047

[228] Y. Ding , B. Xiao , J. Li , Q. Deng , Y. Xu , H. Wang , D. Rao , J. Phys. Chem. C 2019, 123, 3353.

[229] Z. Huang , X. Han , X. Cui , C. He , J. Zhang , X. Wang , Z. Lin , Y. Yang , J. Mater. Chem. A 2020, 8, 5882.

[230] M. Wang , H. Xu , Z. Yang , H. Yang , A. Peng , J. Zhang , J. Chen , Y. Huang , X. Li , G. Cao , ACS Appl. Mater. Interfaces 2019, 11, 41363. 31599565 10.1021/acsami.9b14098

[231] P. Zhang , D. Wang , Q. Zhu , N. Sun , F. Fu , B. Xu , Nano‐Micro Lett. 2019, 11, 81. 10.1007/s40820-019-0312-y PMC7770671 34138047

[232] Z. Wang , J. Sha , E. Liu , C. He , C. Shi , J. Li , N. Zhao , J. Mater. Chem. A 2014, 2, 8893.

[233] L. Wang , X. Bi , S. Yang , Adv. Mater. 2016, 28, 7672. 27346391 10.1002/adma.201601723

[234] W. Wang , L. Hu , L. Li , C. Liu , X. Liu , H. Wang , G. Zhai , Electrochim. Acta 2023, 449, 142239.

[235] L. Wang , X. Zhang , Y. Xu , C. Li , W. Liu , S. Yi , K. Wang , X. Sun , Z. Wu , Y. Ma , Adv. Funct. Mater. 2021, 31, 2104286.

[236] M. R. Islam , S. Afroj , N. Karim , ACS Nano 2023, 17, 18481. 37695696 10.1021/acsnano.3c06181 PMC10540263

[237] F. Haque , T. Daeneke , K. Kalantar‐zadeh , J. Z. Ou , Nano‐Micro Lett. 2018, 10, 23. 10.1007/s40820-017-0176-y PMC6199073 30393672

[238] R. Wang , J. Huang , X. Zhang , J. Han , Z. Zhang , T. Gao , L. Xu , S. Liu , P. Xu , B. Song , ACS Nano 2022, 16, 3593. 35212217 10.1021/acsnano.2c01064

[239] Z. Du , C. Wu , Y. Chen , Z. Cao , R. Hu , Y. Zhang , J. Gu , Y. Cui , H. Chen , Y. Shi , J. Shang , B. Li , S. Yang , Adv. Mater. 2021, 33, 2101473. 10.1002/adma.202101473 34365658

[240] J. Zhou , Q. Tao , B. Ahmed , J. Palisaitis , I. Persson , J. Halim , M. W. Barsoum , P. O. Å. Persson , J. Rosen , Chem. Mater. 2022, 34, 2098.

